# Plakophilin 3 Is Involved in Basal Body Docking in Multiciliated Cells

**DOI:** 10.3390/ijms26115381

**Published:** 2025-06-04

**Authors:** Panagiota Louka, Chrysovalantou Kyriakou, Ioanna Diakourti, Paris Skourides

**Affiliations:** Department of Biological Sciences, University of Cyprus, P.O. Box 20537, 2109 Nicosia, Cyprus; valantookyriakou@gmail.com (C.K.); idiakourti@gmail.com (I.D.)

**Keywords:** plakophilin 3, multiciliated cells, basal body organization, *Xenopus*

## Abstract

Multiciliated cells generate fluid flow along epithelial surfaces, and defects in their development or function cause primary ciliary dyskinesia. The fluid flow is generated by the coordinated beating of motile cilia, which are microtubule-based organelles. The base of each cilium, the basal body, is anchored to the apical cell membrane and surrounded by a dense apical cytoskeleton of actin, microtubules, and intermediate filaments. Several cell adhesion proteins play a role in the connection of the basal body to the apical cytoskeleton. Here, we show that the desmosomal protein plakophilin3, a member of the armadillo family of proteins, localizes to the striated rootlet in *Xenopus laevis* multiciliated cells. Knockdown of plakophilin 3 leads to significant defects in cilia-generated fluid flow and basal body docking. These defects are cell-autonomous and independent of cell intercalation and gross changes in the actin cytoskeleton. These findings suggest a crucial role for PKP3 in basal body apical migration and docking in multiciliated cells, highlighting a novel connection between desmosomal proteins and ciliary function.

## 1. Introduction

Multiciliated cells (MCCs) are differentiated cells found in the brain ventricles, the airway, and the female and male reproductive systems. They are characterized by the presence of hundreds of motile cilia on their surface, which are essential for generating fluid flow along epithelial surfaces. Defects in MCC development or function cause primary ciliary dyskinesia, a debilitating human disease [1,2]. *Xenopus* has a mucociliary epidermis analogous to the mucociliary epidermis in the human trachea, and it is an ideal system for studying MCC development and function.

Motile cilia are microtubule-based organelles that are templated by basal bodies, which are modified centrioles. MCCs generate hundreds of basal bodies de novo [3]. This takes place deep inside the cell, and basal bodies migrate to the apical surface, where they dock at the membrane and facilitate the formation of cilia. Defects in the process of basal body apical migration and docking consequently lead to defects in the formation of cilia and the function of MCCs. The mechanisms that control basal body apical migration and docking in vertebrates are not well understood. So far, only the actin–myosin cytoskeleton has been implicated in this process [4,5,6]. Disruption of the actin cytoskeleton using cytochalasin D impairs basal body migration and docking in MCCs [4]. Proteins that regulate the actin cytoskeleton, such as RhoA, are enriched at basal bodies, and defects in their localization or activation lead to impaired basal body migration [7]. During apical migration, basal bodies are surrounded by a network of actin cables that connect them to the cell cortex, and defects in the formation of these cables correlate with basal body migration and docking defects [8].

In *Xenopus*, MCCs are specified in the deep layer of the epidermis and intercalate to the outer layer [9,10]. Basal body apical migration and docking coincide with cell intercalation, and intercalation defects are often associated with basal body docking defects. Two waves of basal body amplification take place in *Xenopus*, one before and one after cell intercalation [11]. These two pools of basal bodies migrate to the apical surface at different times. The basal bodies that are generated before cell intercalation migrate and dock to the apical cell membrane before intercalation and surface area expansion have been completed [11]. MCCs expand their surface area using a cell-autonomous actin-based mechanism [12]. Interestingly, the number of basal bodies generated by a cell correlates with the cell surface area [11].

Basal bodies are anchored to the apical cell membrane via distal appendages [13] and are embedded in a dense apical cytoskeleton of actin, microtubules, and intermediate filaments [14]. To generate effective flow, cilia and their basal bodies must be properly spaced and polarized. The actin cytoskeleton is important for the spacing of basal bodies at the apical surface, whereas the microtubule cytoskeleton is implicated in basal body polarity [4,5,6,7,15,16]. Basal bodies have two conserved accessory structures that point in opposite directions: the basal foot and striated rootlet. The basal foot acts as a microtubule organizing center [17,18] and points towards the direction of ciliary beating (the direction of the power stroke) [19,20,21,22]. The striated rootlet is attached to one side of the basal body, pointing opposite to the direction of ciliary beating. The rootlet diameter is the widest, where it connects to the basal body, and it tapers out as it moves away from the basal body [23]. Rootletin is likely the sole structural component of rootlets. Rootletin polymerization leads to the characteristic rootlet structure made of striated fibers [24], and loss of rootletin leads to loss of the rootlet [25,26,27]. The molecular composition and function of the rootlet in vertebrate motile cilia is not well understood. Intriguingly, in rootletin knockout mice, the rootlet is absent in MCCs, but MCCs in the trachea are ciliated [25]. This suggests that the rootlet is not essential for basal body migration and ciliogenesis in the mouse trachea.

The striated rootlet is linked to the actin cytoskeleton through ciliary adhesions (CAs) [28]. Loss of the CA protein FAK and the actin regulator Fhod3, which also localizes near ciliary adhesions, affects the connection of the basal body to the actin cytoskeleton and leads to basal body docking and ciliogenesis defects [28,29]. In the proteome interactome database BioGRID [30], we noticed that all known CA components interact with junctional plakoglobin (JUP), a component of desmosomes. JUP is not known to associate with focal adhesions, and the known CA proteins are not known to associate with desmosomes, suggesting that they might interact in a different subcellular compartment. Intriguingly, JUP is detected in some ciliary proteomes [31,32], raising the possibility that, similar to focal adhesion proteins, desmosomal proteins may also have a specialized function in ciliated cells. Indeed, desmoplakin (DSP), plakophilins (PKP), and plakoglobin are all detected in the proteome of photoreceptors from wild-type mice, and interestingly, they are missing from the photoreceptor proteome of rootletin knockout mice [31]. DSP is a core desmosomal protein that links desmosomes to intermediate filaments through its interaction with plakophilins and plakoglobin. DSP and plakophilin 3 (PKP3) are important for the development of the epidermis in *Xenopus* which is consistent with their role in desmosomes [33,34]. In addition, the downregulation of DSP affects cell intercalation and ciliogenesis. However, it is still unclear if the ciliogenesis defect is secondary to the intercalation defects [34]. The same is true for PKP3, which was also shown to affect ciliogenesis and MCC density [33].

Here, using live imaging, targeted gene expression, and morpholino-mediated knockdown, we demonstrate the novel localization of PKP3 at the striated rootlet of basal bodies in *Xenopus* MCCs. The sole PKP expressed in Xenopus MCCs is PKP3, and its downregulation led to defective cilia-generated fluid flow and basal body docking. Importantly, we show that the defect in basal body docking is cell-autonomous and independent of cell intercalation and the actin cytoskeleton.

## 2. Results

### 2.1. Plakophilin 3 Localizes at the Striated Rootlet

**Figure 1:**
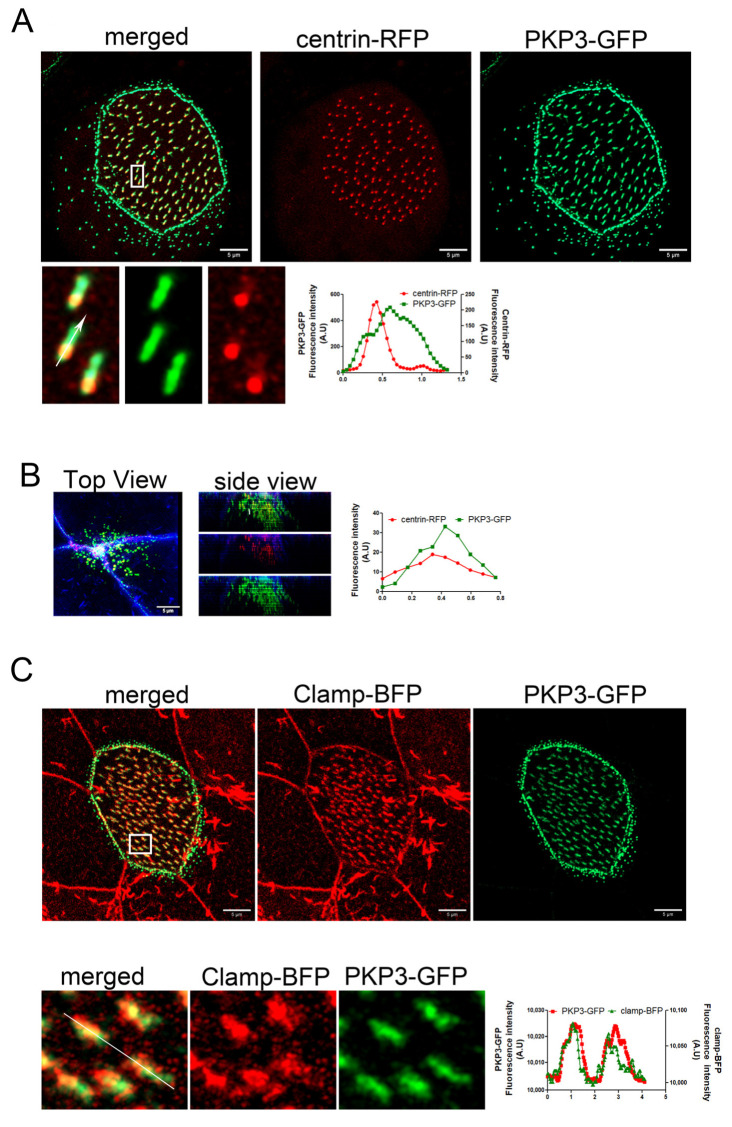
PKP3 localizes at the striated rootlet in *Xenopus* MCCs. Maximum intensity projection (MIP) confocal image of centrin-RFP (red) and PKP3-GFP (green) in *Xenopus* mature MCCs (**A**) and intercalating cells (**B**). Graphs show the fluorescence intensity profile for centrin-RFP and PKP3-GFP along the white arrow in the zoomed-in image. (**C**) MIP confocal image of a mature MCC expressing PKP3-GFP (green) and clamp-BFP (red). The graph shows the fluorescence intensity for PKP3-GFP and clamp-BFP along the white line shown in the zoomed-in image.

Plakophilins are a family of armadillo repeat proteins that connect desmosomal cadherins to desmoplakin and intermediate filaments (PKP1–4). In addition to their structural function within desmosomes, plakophilins (PKPs) serve as pivotal regulators of diverse signaling cascades, such as transcriptional regulation, cellular growth, proliferation, and migration [35]. The mechanisms that enable plakophilins (PKPs) to integrate adhesive functions with signaling activities and engage in interactions with numerous binding partners are still not well established. *Xenopus* MCCs express only PKP3 [36]. In Xenopus, there are two genes that encode PKP3 homologs: PKP3L and PKP3S. We did not find a commercially available antibody that recognizes the *Xenopus* PKP3; therefore, we used protein overexpression to assess the localization of PKP3 in MCCs. The *Xenopus* PKP3L was cloned in a vector for C-terminal GFP tagging. Expression of this construct in *Xenopus* showed a characteristic desmosomal localization and an association with basal bodies in MCCs (Figure S1). However, this construct was unstable and highly degraded. Thus, to examine the localization of PKP3 in MCCs, we used a PKP3 construct that was previously characterized [37]. This construct encodes human PKP3 fused to GFP. Multiple sequence alignment of the Xenopus and human PKP3 proteins shows that they are approximately 60–62% identical, and pairwise alignment showed 70% similarity (Figure S2). We expressed human PKP3a fused to GFP along with centrin-RFP to determine its localization in MCCs. Live imaging of MCCs revealed localization of PKP3 in a desmosome-like pattern along the apical and lateral cell membrane and an additional enrichment near basal bodies in both intercalating and mature MCCs (Figure 1A,B). The PKP3-GFP basal body-associated signal is present in MCCs irrespective of the expression levels of the GFP construct (Figure S3). Also, MCCs that express PKP3-GFP do not have any phenotypes related to basal body organization, docking, and ciliogenesis. Thus, the expression of exogenous PKP3-GFP is unlikely to cause an overexpression phenotype in our experiments. The PKP3 signal is asymmetric with respect to basal bodies, suggesting that it localizes to a polarized basal body accessory structure. Expression of PKP3-GFP and the rootlet-associated protein clamp-BFP shows that PKP3 co-localizes with clamp (Figure 1C).

**Figure 2:**
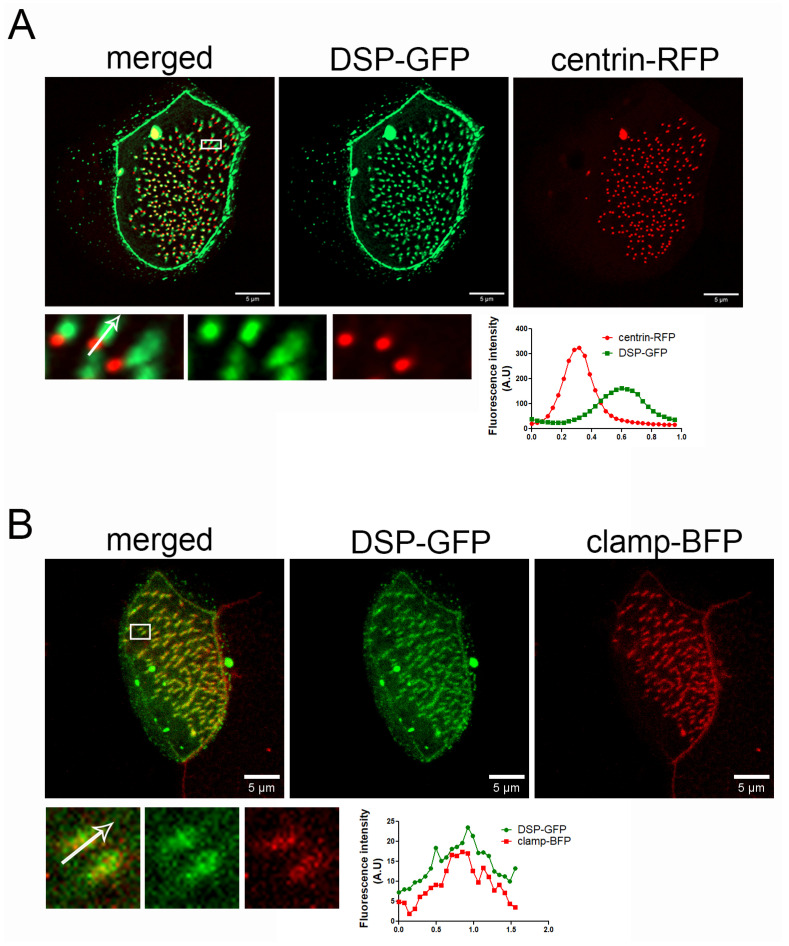
DSP localizes at the striated rootlet in *Xenopus* MCCs. (**A**) MIP super-resolution confocal image of centrin-RFP (red) and DSP-GFP (green) in *Xenopus* mature MCCs. The graph shows the fluorescence intensity of centrin-RFP and DSP-GFP along the white arrow in the zoomed-in image. (**B**) MIP confocal image of a MCC expressing DSP-GFP (green) and clamp-BFP (red). The graph shows the fluorescence intensity of clamp-BFP and DSP-GFP along the white arrow in the zoomed-in image.

PKPs interact with and enhance the recruitment of desmoplakin (DSP) at desmosomes. We examined if DSP shows a similar localization pattern to PKP3 in MCCs. We expressed human DSP-GFP (67% identity and 82.5% similarity with Xenopus DSP, Figure S4) with centrin-RFP and imaged embryos live. DSP-GFP shows the characteristic desmosomal pattern and an enrichment near basal bodies (Figure 2A). Similar to PKP3, the DSP signal is asymmetric with respect to basal bodies. Imaging of fixed MCCs that express DSP-GFP and clamp-BFP shows that DSP co-localizes with clamp (Figure 2B).

These data reveal a novel localization for PKP3 and DSP at the striated rootlet in MCCs.

### 2.2. PKP3 Does Not Co-Localize with Keratin 8 Intermediate Filaments, Actin, or Microtubules

**Figure 3:**
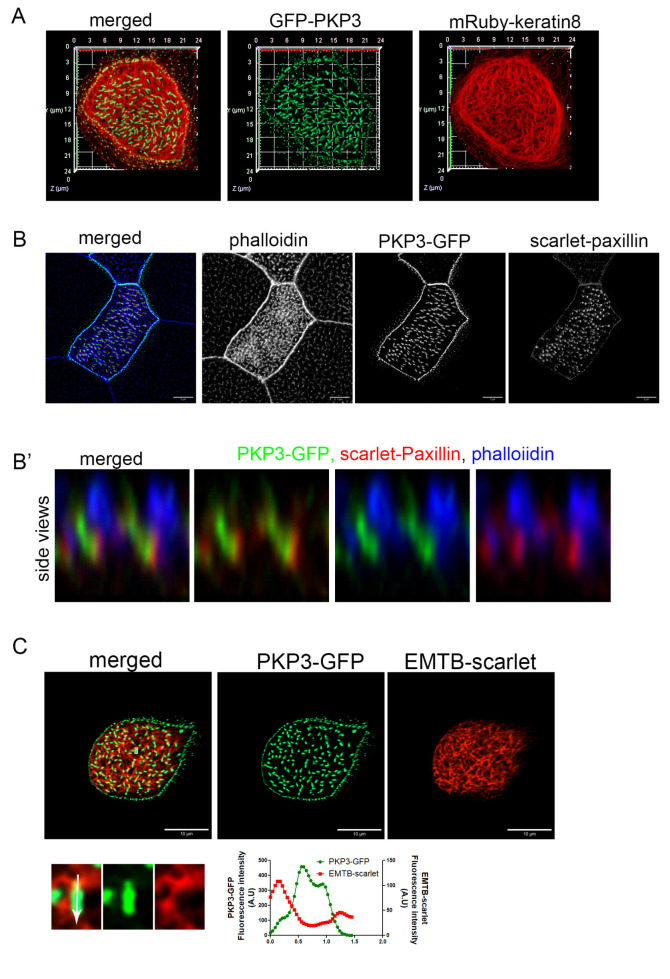
PKP3 localization with respect to keratin 8 intermediate filaments, actin, and microtubules. (**A**) Three-dimensional image of mRuby-keratin 8 (red) and PKP3-GFP (green) in a mature MCC. (**B**) MIP confocal image of a mature MCC labeled with phalloidin (blue), scarlet–paxillin (red), and PKP3-GFP (green). (**B’**) Side views of actin, paxillin, and PKP3. (**C**) Confocal image of EMTB-scarlet and PKP3-GFP in a developing MCC. A zoomed-in image shows the area marked by a white box. The graph shows the fluorescence intensity of EMTB-scarlet and PKP3-GFP along the white arrow in the zoomed-in image.

At desmosomes, PKP3 interacts with DSP, which links desmosomes to intermediate filaments. The presence of both PKP3 and DSP at the rootlet raised the possibility that these proteins may link the rootlet to intermediate filaments. Keratin 8 filaments are enriched in the apical cytoskeleton in MCCs in mice [38]. Here, we show that mRuby-Keratin 8 is also enriched in the apical cytoskeleton in *Xenopus* MCCs and that it overlaps with the apical actin and forms a mesh-like network sub-apically around the sub-apical actin foci (Figure S5). In MCCs, PKP3-GFP localizes near the end of keratin 8 filaments at the lateral cell membrane; however, we do not see any co-localization between PKP3 and keratin 8 at the rootlet (Figure 3A). We then examined the localization of PKP3 with respect to the actin and microtubule cytoskeleton. To visualize the actin cytoskeleton, embryos were stained with the F-actin-binding protein phalloidin. To visualize the cytoplasmic microtubules, embryos were injected with the well-defined microtubule reporter, EMTB [39]. PKP3 was detected in close proximity to the sub-apical actin near its basal end (Figure 3B) and in close proximity to the EMTB-scarlet near the apical end of the PKP3 signal (Figure 3C). The rootlet is linked to the sub-apical actin through CAs. We observed that the PKP3 signal partially overlaps with the CA component scarlet–paxillin, which links the rootlet to the sub-apical actin (Figure 3B). These data further refine the rootlet localization of PKP3, showing that it localizes near CAs and is unlikely to link the rootlet to the cytoskeleton.

### 2.3. Downregulation of PKP3 Affects the Function of MCCs

**Figure 4:**
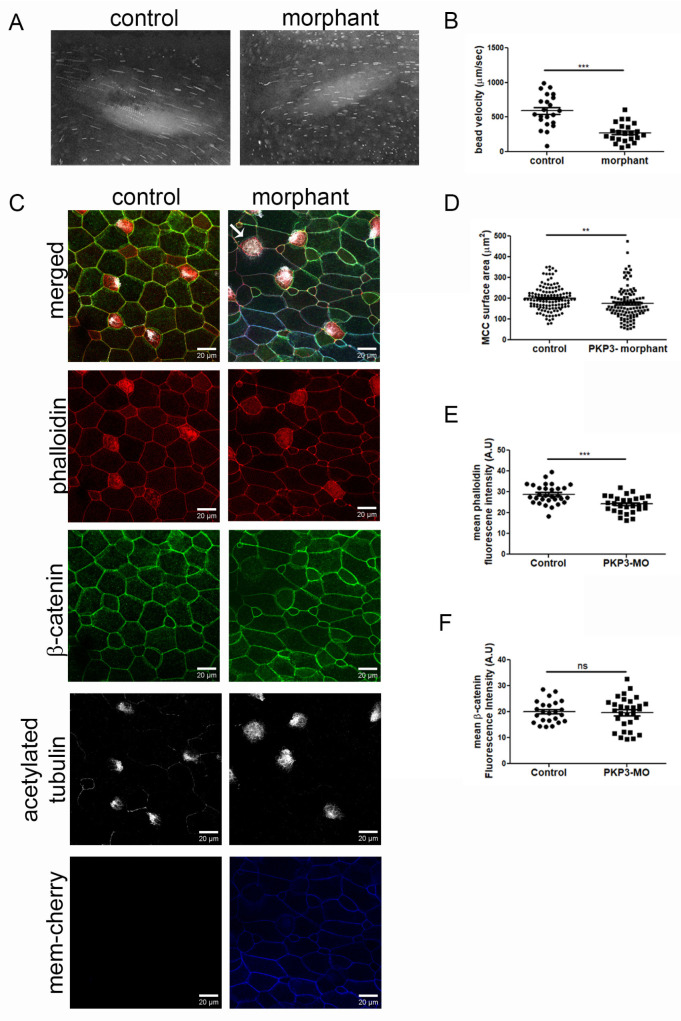
Downregulation of PKP3 affects the function of MCCs. (**A**) Images from movies 1 and 2 show the bead trajectory in 10 frames. (**B**) Quantification of bead velocity (mean control 592 μm/s SEM: 50, n = 22 embryos and mean morphant 275 μm/s, SEM: 29, n = 23 embryos). (**C**) Confocal MIP images from control and morphant embryos (red: phalloidin; green: β-catenin; white: acetylated tubulin; and blue: morpholino tracer mem-cherry). The arrow points to an MCC in the morphant that lacks cilia on its surface. (**D**) Quantification of MCC surface area (control: 200 μm^2^; SEM: 5.2; n = 126 MCCs; and morphant: 174 μm^2^, SEM: 7.5, n = 110 MCCs). (**E**) Phalloidin mean fluorescence intensity (control = 29 A.U, SEM: 0.84, n = 30 MCCs and morphant = 24 A.U, SEM: 0.72, n = 30 MCCs). (**F**) β-catenin mean fluorescence intensity (control = 20 A.U, SEM: 0.86, n = 24 MCCs and morphant = 20 A.U, SEM: 1.1, n = 30 MCCs) (*t*-test: ** *p* < 0.01, *** *p* < 0.001, ns: not significant).

To address the function of PKP3 in MCCs, we used a previously characterized translation-blocking morpholino [33]. Through dose–response experiments, we identified a morpholino dose that does not elicit any defects in skin integrity, such as fragility, as previously reported [33]. Embryos were injected with the morpholino and mem-cherry as a tracer in the two ventral blastomeres at the four-cell stage to deliver the morpholino to the entire epidermis. Quantification of fluorescence bead velocity as they move across the tadpole body shows a significant reduction in cilia-generated fluid flow (Figure 4A,B. movies 1 and 2). Immunofluorescence analysis of control and morphant embryos showed a significant decrease in phalloidin fluorescence intensity and MCC surface area in the morphants (Figure 4C,D). We did not detect any gross cell–cell junction defects, as indicated by β-catenin staining (Figure 4E). These data show that PKP3 is important for the function of MCCs.

**Figure 5:**
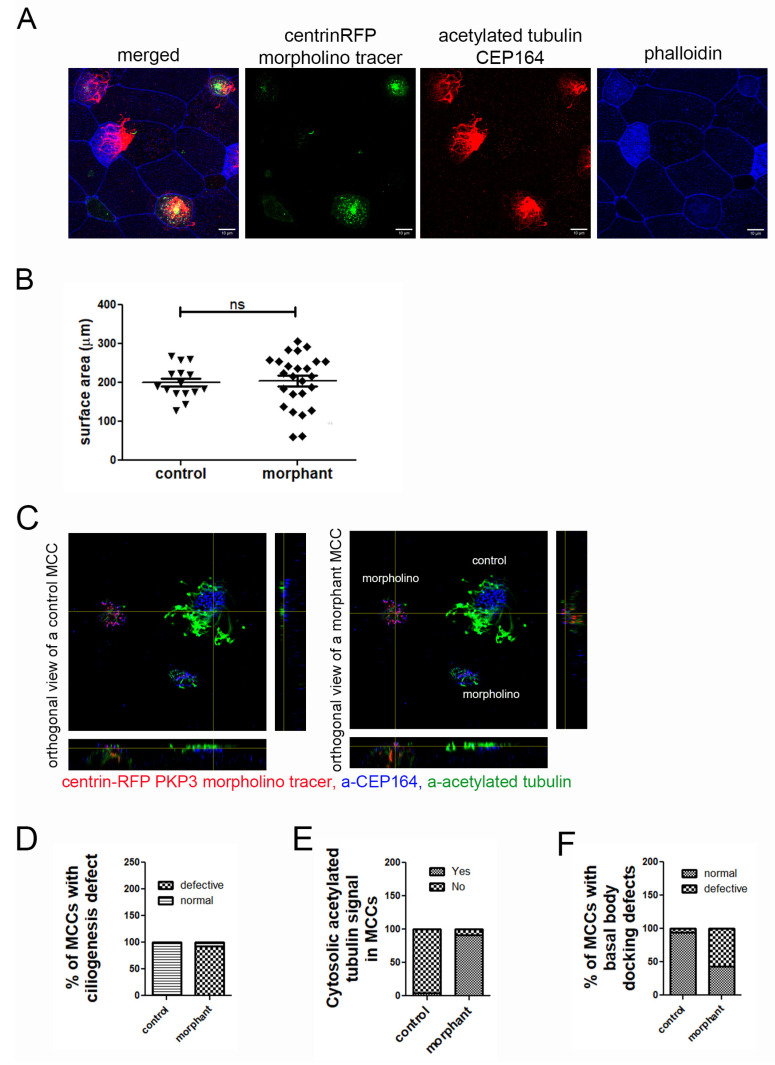
Downregulation of PKP3 elicits a cell-autonomous effect on ciliogenesis and basal body docking. (**A**) MIP confocal images of the epidermis of mosaic morphant embryos stained for acetylated tubulin and labeled with phalloidin. (**B**) Quantification of the surface area of control and morphant MCCs in mosaic morphant embryos (mean control = 200 μm^2^, standard deviation = 11, n = 15 MCCs, mean morphant = 204 μm^2^, standard deviation = 14, n = 25 MCCs, ns: not significant). (**C**) Orthogonal views of control and morphant MCC on a tadpole epidermis with mosaic expression of PKP3 morpholino (blue: a-CEP164; green: acetylated tubulin; red: centrin-RFP morpholino tracer). (**D**) Quantification of the ciliogenesis defect (n = 284 control and 222 morphant MCCs; chi-square, *p* < 0.001). (**E**) Quantification of the number of control and morphant MCCs that have acetylated tubulin signal deep in the cytosol (n = 88 control and 77 morphant MCCs; chi-square, *p* < 0.0001). (**F**) Quantification of basal body docking defect (n= 80 control and 133 morphant cells; chi-square test, *p* < 0.001).

Despite the lack of overt skin fragility in PKP3 morphants, it is still possible that the entire mucociliary epithelium is impacted in our experiments, leading to secondary defects in MCCs since epithelial tension and mechanics are critical regulators of MCC differentiation and intercalation. To exclude the possibility that defects in cell–cell contacts contribute to the effect of PKP3 on MCC function, we generated mosaic morphant embryos. Embryos were injected in two ventral blastomeres at the 16-cell stage with the morpholino and centrin-RFP as tracer. In these embryos, morphant MCCs are surrounded by normal cells. The embryos were stained with antibodies against CEP164 and acetylated tubulin to mark MCCs and with phalloidin to mark the F-actin network and use the cortical actin signal to quantify the MCC surface area. Interestingly, morphant MCCs show increased variability in their surface area compared to controls. Despite this variability, we did not observe any significant defects in the surface area of MCCs in mosaic morphant embryos (Figure 5A,B).

To determine the effect of PKP3 on MCCs, we stained mosaic morphant embryos that were injected with centrin-RFP as a morpholino tracer, with antibodies against acetylated tubulin (Figure 5C, green) and CEP164 (Figure 5C, blue), which are commonly used to mark cilia and basal bodies, respectively. We quantified the number of control and morphant MCCs that had a ciliogenesis defect. Morphant MCCs that looked different from control MCCs in terms of the apical enrichment of acetylated tubulin were scored as defective, and those that looked similar to control MCCs were scored as normal. Our data show that most morphant MCCs had ciliogenesis defects (Figure 5C,D). During this analysis, we noticed that unlike in control MCCs in which acetylated tubulin is enriched apically, in morphant MCCs, acetylated tubulin was enriched basally (Figure 5E). The presence of acetylated tubulin in the cytosol is often observed in MCCs with ciliogenesis defects.

The ciliogenesis defect could be caused by defects in axoneme elongation or basal body docking. To dissect the two, we quantified the number of control and morphant MCCs that have basal body docking defects. When viewed from the side (orthogonal projection), docked basal bodies form an almost straight line. In contrast, non-docked basal bodies are spread along the z-axis (or apicobasal axis) of the cell. MCCs in which basal bodies did not align in an almost straight line were classified as defective in terms of basal body docking. Using this scoring criterium, we found that significantly more morphant MCCs exhibited basal body docking defects compared to control MCCs (Figure 5C,F).

Next, given that PKP3 localizes to the rootlet, we examined if the localization of the rootlet-associated protein clamp is affected in the morphant. The striated rootlet angle changes with respect to the apical surface as the basal bodies become polarized in the cells [29]. In immature MCCs, the rootlet projects orthogonal to the nucleus, whereas as basal bodies become polarized, the rootlet tilts towards the apical membrane, and it can be viewed as an elongated polarized structure in 2D image projections. We used the rootlet-associated protein clamp as a proxy for the rootlet. In orthogonal projections of MCCs expressing clamp-GFP and centrin-RFP, the clamp signal projects basally from the basal body in both control and morphant MCCs (Figure S6).

Taken together, these data show that PKP3 downregulation elicits a cell-autonomous effect on basal body docking and ciliogenesis.

### 2.4. Basal Body Docking Defect in PKP3 Morphant MCCs Is Independent of MCC Intercalation

**Figure 6:**
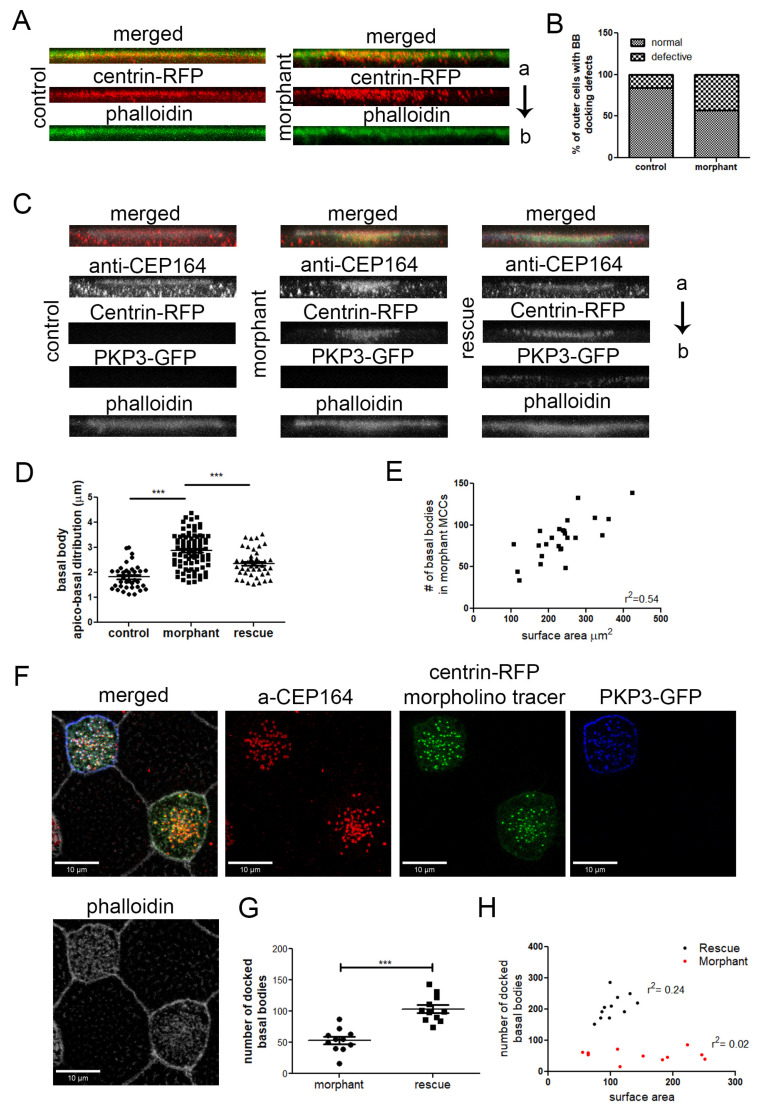
The effect of PKP3 on basal body docking is independent of cell intercalation. (**A**) Side views of z-projections of control and morphant outer cells induced with multicilin to differentiate to MCCs (red: centrin-RFP; green: phalloidin). (**B**) Quantification of the basal body docking defect in outer MCCs (n = 80 control and 133 morphant cells; chi-square, *p* < 0.001). (**C**) Side view of z-projections of MCCs from control, morphant embryos, and embryos injected with the PKP3 morpholino and hPKP3-GFP (red: anti-CEP164; green: centrin-RFP; blue: hPKP3-GFP; white: phalloidin). (**D**) Quantification of the apicobasal distribution of basal bodies in MCCs in control (n = 36 cells), morphant (n = 86 cells), and rescue embryos (n = 44 cells) (one-way ANOVA and Tukey’s multiple comparison test). (**E**) The total number of basal bodies in morphant MCCs is shown against the MCC surface area. (**F**) Confocal image showing the docked basal bodies in a morphant and rescue MCC. (**G**) Quantification of the number of docked basal bodies in morphant and rescue MCCs (morphant: 54, standard deviation: 19, n = 11 MCCs, control: 104, standard deviation: 21, n = 11 MCCs; *t*-test, *** *p* < 0.001). (**H**) Correlation of docked basal bodies to the MCC surface area (μm^2^) in morphant and rescue MCCs. (a: apical and b: basal end of the cell).

Basal bodies are generated deep inside the cell and migrate apically during MCC intercalation. To exclude the possibility that the basal body docking defect is linked to potential intercalation defects, we induced the differentiation of outer cells to MCCs with an expression of multicilin [40]. We used the same scoring criteria for basal body docking defects as described before. Significantly more morphant outer MCCs had basal body docking defects compared to control outer MCCs (Figure 6A,B).

To determine the specificity of this phenotype, we examined the ability of PKP3-GFP to rescue the basal body docking and ciliogenesis defects. Embryos were injected in the two ventral blastomeres at the 4-cell stage with PKP3-GFP and in two out of four ventral blastomeres at the 16-cell stage with PKP3 morpholino and centrin-RFP as a tracer. Embryos were stained with anti-CEP164 to label the basal body distal appendages and mark the position of basal bodies and with phalloidin to mark the cell boundaries. The CEP164 signal includes some background signal as well, which is present in both non-MCCs and MCCs (Figure S7). The scoring criteria for basal body docking defects described above were too broad to distinguish between various degrees of rescue. Therefore, we quantified the distance in the z-axis from the most apical to the most basal basal body using side projections of MCCs. The length of basal body distribution in the z-axis is significantly increased in morphant MCCs compared to control cells, and this is partially rescued in morphant cells that express PKP3-GFP (Figure 6C,D). We also quantified the number of docked basal bodies in morphant and rescue MCCs. For this quantification, we used optical sections from confocal images at the level of the apical membrane marked by a strong cortical phalloidin signal. Morphant MCCs that express PKP3-GFP had significantly more docked basal bodies compared to morphant MCCs (Figure 6G).

Next, we tested the possibility that the rescue in the number of docked basal bodies may be due to an increase in the total number of basal bodies. The total number of basal bodies scales with the surface area [11]. Quantification of the total number of basal bodies and of the cell surface area shows that the basal body number scales with the cell surface area in morphant MCCs (Figure 6E). Therefore, PKP3 does not affect the mechanisms that control basal body amplification. The number of docked basal bodies in the morphants does not correlate with the cell surface area, which is expected when basal body docking is impaired (Figure 6H). The correlation between docked basal bodies and MCC surface area is improved in morphant MCCs that express PKP3-GFP (Figure 6H). Although the basal body docking defect is partially rescued, we failed to quantify changes in ciliogenesis between morphant and rescue MCCs. Several factors could contribute to the lack of rescue of the ciliogenesis defect, including differences between the human and Xenopus PKP3 and technical limitations due to the fact that morphant MCCs are partially ciliated, and small changes in cilia density may have gone unnoticed.

Taken together, our data show that the basal body defect in morphant MCCs is specific to PKP3 downregulation, and it is independent of cell intercalation.

### 2.5. Basal Body Docking Defect Is Independent of the Actin Cytoskeleton

**Figure 7:**
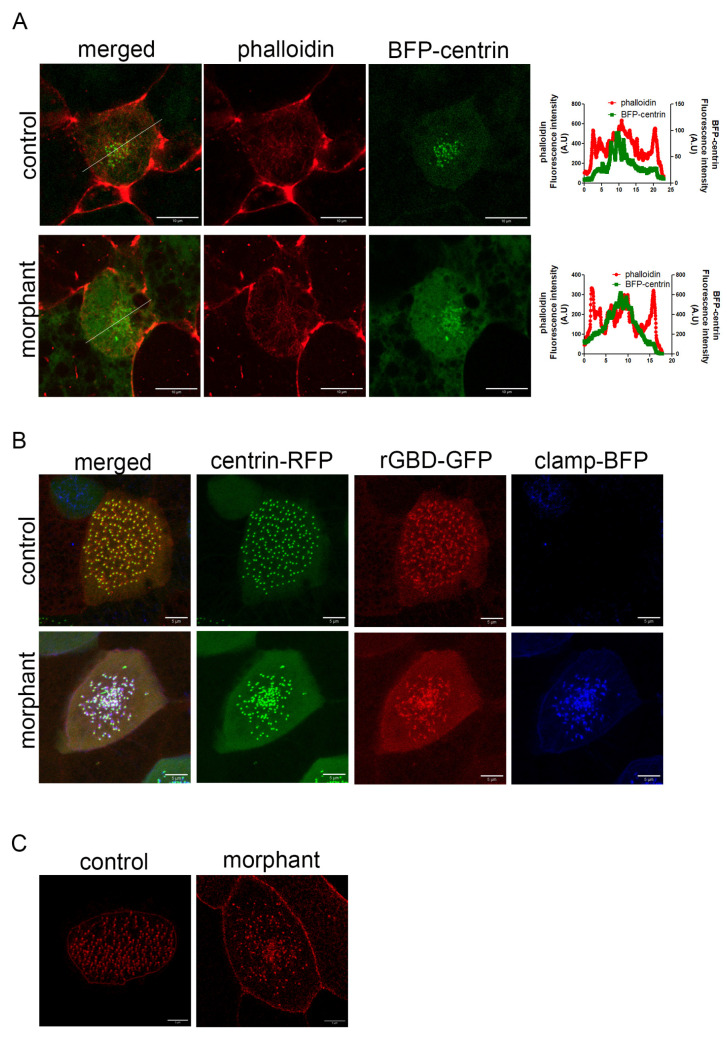
Downregulation of PKP3 does not affect the actin cytoskeleton. (**A**) Single optical section of MCCs during intercalation (green: BFP-centrin; red: phalloidin). Graphs show the fluorescence intensity for phalloidin and centrin across the white line shown in the merged image. (**B**) Localization of active RhoA sensor (rGBD-GFP) in control and morphant MCCs in a mosaic morphant embryo (green: centrin-RFP; red: rGBD-GFP; blue: clamp-BFP as the morpholino tracer). (**C**) MIP confocal image of a representative control and morphant MCC expressing scarlet–paxillin.

The mechanisms that control basal body migration and docking are not well understood. So far, only the actin cytoskeleton has been implicated in this process. Specifically, actin cables surround basal bodies during migration, and defects in the formation of these cables correlate with defects in migration and docking [8]. To examine if the actin cables are affected in morphants, we stained embryos during the process of MCC intercalation with phalloidin. These embryos were injected with BFP-centrin. Actin cables were readily detectable in both control and morphant MCCs (Figure 7A). Analysis of the fluorescence intensity of phalloidin and centrin across the cell shows enrichment of phalloidin at cell borders and around the centrin signal (Figure 7A). Thus, actin enrichment around basal bodies and the formation of actin cables during intercalation are likely unaffected in the morphants. Note that differences in the expression levels of BFP-centrin lead to variation in the BFP background signal between control and morphant MCCs (Figure 7A,B).

Next, we examined if RhoA, a protein that regulates the actin cytoskeleton and is important for basal body migration and organization, is affected by PKP3 downregulation. Active RhoA recruitment to basal bodies is necessary for basal body apical migration [7]. We injected embryos in the two ventral blastomeres at the 4-cell stage with an active RhoA biosensor (rGBD-GFP) [41] along with centrin-RFP, and the same embryos were injected in two out of four ventral blastomeres at the 16-cell stage with the PKP3-morpholino and clamp-BFP as a tracer. In maximum-intensity projections of confocal images of morphant MCCs, the basal bodies appeared clustered in the center of the cell. This is because basal bodies migrate as a cluster, and when they fail to dock, they remain clustered below the membrane, as seen in morphant MCCs. Interestingly, rGBD-GFP is recruited to the basal bodies in both control and morphant MCCs (Figure 7B), showing that the effect of PKP3 on basal body docking is not mediated through RhoA activation near basal bodies.

PKP3 at the rootlet localizes near CAs, and defects in CAs affect the connection of the rootlet to the actin cytoskeleton and lead to basal body docking defects [28]. To address whether the effect of PKP3 on basal body docking stems from disruption of the CA complex, we examined the localization of the CA protein paxillin in control and morphant MCCs. Scarlet paxillin was enriched at the medioapical surface of control and morphant MCCs, consistent with its localization to the rootlet (Figure 7C). This suggests that the effect of PKP3 on basal body docking is not mediated through paxillin.

Taken together, our data support that the role of PKP3 in basal body docking is likely mediated through an actin-independent mechanism.

### 2.6. Downregulation of PKP3 Does Not Affect the Recruitment of DSP to Basal Bodies

**Figure 8:**
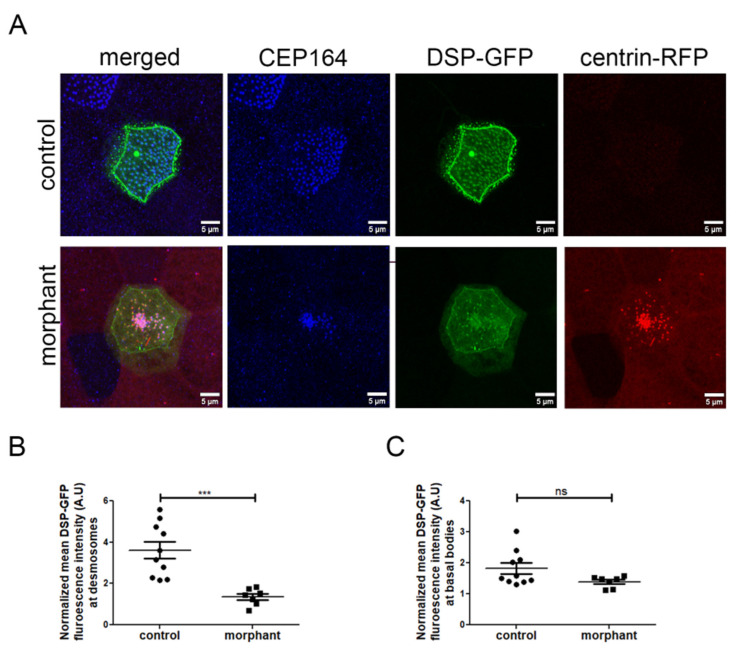
Downregulation of PKP3 affects the desmosomal but not the rootlet localization of DSP. (**A**) MIP confocal images of control and morphant MCCs expressing DSP-GFP (blue: CEP164, green: DSP-GFP, red: centrin-RFP(morpholino tracer)). (**B**,**C**) The DSP-GFP fluorescence intensity was normalized to the cytosolic levels of DSP-GFP and mean DSP-GFP at desmosomes (**B**) and basal bodies (**C**) was quantified (control: mean normalized DSP-GFP signal at desmosomes 3.6 (A.U) and basal body 1.8 (A.U), n = 10 MCCs and morphant: mean normalized DSP-GFP signal at desmosomes 1.36 (A.U) and basal body 1.4 (A.U), n = 7 MCCs; *t*-test, *** *p* < 0.001, ns: not significant).

To better understand how PKP3 may affect basal body docking, we examined its effect on the localization of its known interactor DSP. Embryos were injected in the two ventral blastomeres at the 4-cell stage with DSP-GFP and in two out of four ventral blastomeres at the 16-cell stage with the morpholino and centrin-RFP as a tracer. Embryos were stained against CEP164 to mark the position of basal bodies in control MCCs. We quantified the mean fluorescence intensity of DSP at desmosomes and the rootlet in mosaic morphant embryos. The fluorescence intensity was normalized to the mean cytosolic DSP signal to account for differences in DSP-GFP expression levels. As expected, we observed a decrease in the DSP signal at desmosomes in morphant MCCs (Figure 8A,B). Intriguingly, the DSP signal near basal bodies was not significantly affected in morphant compared to control MCCs (Figure 8A,C). Therefore, it is likely that different mechanisms control the localization of DSP at desmosomes and basal bodies. This also raises the possibility that defects at desmosomes may contribute to the basal body docking defect in PKP3 morphant embryos.

Another way to better understand the function of PKP3 at basal bodies is to study its interactome near basal bodies. However, this is technically very challenging to achieve through affinity tag pull-down or proximity labeling experiments because the PKP3 desmosomal interactors would likely mask the identification of PKP3 basal body interactors. Alternatively, we explored the PKP3 interactome using the publicly available proteome interaction database BioGRID [30]. We filtered the 153 PKP3 interactors for proteins with known function in cilia and centrioles and proteins that have been identified in ciliary proteomes. This led to the identification of 18 putative cilia-related PKP3 interactors (Table S1). This includes proteins with known basal body localization and function in MCCs such as WDR5 [42], disheveled [43], JNK [44], and the kinesin2 subunit Kif3A [45,46,47]. Further studies are needed to validate these possible interactions.

## 3. Discussion

This study reveals a novel and essential role for PKP3 in basal body organization in *Xenopus* MCCs. We demonstrate the specific localization of PKP3, along with DSP, at the striated rootlet, a finding that expands our understanding of the molecular composition of this crucial ciliary structure. This localization is further supported by the detection of both proteins in the proteome of photoreceptors and the absence from the proteome of rootletin knockout photoreceptors [31]. While PKP3 and DSP are known components of desmosomes, they also have non-desmosomal functions, and their presence at the rootlet suggests a specialized function in MCCs.

In our experiments, we used a translation-blocking morpholino that has been previously shown [33] to lead to reduced PKP3 expression, which is the only PKP expressed in these cells. PKP3 morphant embryos exhibited reduced fluid flow generation, decreased phalloidin staining, and a smaller MCC surface area. These functional impairments were accompanied by defects in basal body docking and ciliogenesis. Basal bodies in PKP3 morphant MCCs failed to properly migrate and dock at the cell membrane. The apical and subapical actin networks form around docked basal bodies [29]. In morphant MCCs, docked basal bodies are surrounded by phalloidin, showing that F-actin does form around docked basal bodies. Because fewer basal bodies dock in morphant compared to control MCCs, it is not surprising that morphant MCCs have lower levels of phalloidin fluorescence intensity. Thus, the reduced phalloidin signal in morphant cells is likely secondary to the basal body docking defect. Importantly, the docking defects were also observed in mosaic morphant embryos and in outer cells induced to differentiate to MCCs. This demonstrates a cell-autonomous and cell intercalation-independent effect of the loss of PKP3, excluding the possibility that general epithelial defects were indirectly affecting MCC development.

The actin cytoskeleton is important for basal body migration and docking [4,5,6,7,16]. However, our analysis showed no gross disruption of actin cables or changes in the localization of the key actin regulators, paxillin and active RhoA, in PKP3 morphants. This is consistent with data showing that PKP3 at the rootlet is in close proximity to, but does not directly overlap with, the sub-apical actin network. These findings strongly suggest that PKP3’s role in MCCs is distinct from the known mechanisms governing actin-dependent basal body migration. How PKP3 may contribute to basal body migration remains elusive.

Intriguingly, in rootletin mutant mice that lack the rootlet, tracheal MCCs are ciliated, suggesting that basal body apical migration is unaffected [25]. PKP3 co-localizes with centrin as well, and it remains to be examined if loss of the rootlet abolishes PKP3 from basal bodies. Interestingly, DSP does not co-localize with centrin, and its basal body localization is unaffected by the downregulation of PKP3. These data suggest that DSP and PKP3 may not form the same protein complex at the basal body as they do at desmosomes. On the other hand, DSP localization at desmosomes is significantly reduced in morphant MCCs. This raises the possibility that defects at desmosomes may contribute to the observed basal body defects. Further studies are needed to determine the role of PKP3 in desmosome-intermediate filament connection and its contribution towards mechanical resistance/homeostasis and basal body organization in this context.

Our study raises several intriguing questions. While we have shown that PKP3 is essential for proper basal body docking, the precise molecular mechanism by which it exerts this effect remains to be elucidated. Given its localization at the rootlet, PKP3 may be involved in anchoring the basal body to intermediate filaments other than keratin 8. Future studies may examine the PKP3 interactome at the rootlet to dissect its basal body and desmosomal function and examine how PKP3 may contribute to the forces required for apical–basal body migration and docking. Addressing these questions will provide a more comprehensive understanding of the molecular mechanisms governing basal body migration and docking. Furthermore, the observation that PKP3 and DSP, traditionally considered desmosomal proteins, localize at the rootlet highlights the multifunctional nature of these proteins and opens up new avenues of research into the potential crosstalk between cell adhesion and ciliogenesis.

## 4. Materials and Methods

### 4.1. Embryo Manipulations and Microinjections

Adult female *Xenopus laevis* frogs were induced to ovulate by subcutaneous injection of 750 μg/mL of human chorionic gonadotropin hormone. The next day, eggs were collected and fertilized in vitro. Embryos were dejellied using 1.8% cysteine in 1/3MMR and reared in 4% ficoll in 1/3MMR. Microinjections were performed using a glass capillary pulled needle, forceps, a Singer Instruments MK1 micromanipulator (Singer Instruments, Roadwater, UK), and a Harvard Apparatus pressure injector (Holliston, MA, USA). After microinjections, embryos were reared in 4% ficoll for 1–2 h and subsequently transferred to 0.1× MMR. To target MCCs on the epidermis, injections were performed in the two ventral blastomeres of 4- and 8-cell stage embryos. To generate mosaic expression on the epidermis, constructs were injected in two ventral blastomeres of 16-cell stage embryos. Embryos were imaged live at the appropriate developmental stage or fixed in MEMFA and processed for immunofluorescence analysis and imaging.

### 4.2. DNA Constructs and Morpholino Oligonucleotide

The human PKP3-GFP construct was a gift from Dr. Kathleen J. Green (Feinberg School of Medicine, Northwestern University). We used a previously characterized PKP3 translation-blocking morpholino [33]. Uninjected embryos were used as control. Human keratin 8 sequence was amplified from keratin 8 pCDNA3 plasmid (addgene #18063) and cloned using BamHI and KpnI in a pCs105 vector containing an N-terminal mRuby tag. We used SP6 and T7 mMessage mMachine Kit (Invitrogen, Thermo Fisher Scientific, Waltham, MA, USA) to generate capped mRNA for mRuby-keratin 8, EMTB-scarlet (human), centrin-RFP (*Xenopus*), Clamp-BFP (*Xenopus*), and Clamp-GFP (*Xenopus*). *Xenopus* PKP3L was amplified using the following set of primers: the forward primer was 5′-ATGCAGGAAAGTCACTTTCTTATG-3′, and the reverse primer was 5′-TGGATTGAGAAAGTCGTCCTTG-3′ and cloned using NotI and XbaI in pCS108 vector containing a C-terminal GFP tag. Injections were performed in the two ventral blastomeres at the 4-cell stage or two out of four ventral blastomeres at the 16-cell stage. A total of 16 ng morpholino was injected along with a tracer (160 pg centrin-RFP or 200 pg Clamp-GFP); mRNA for mRuby-keratin 8 (100 pg) and EMTB-scarlet (300 pg) were used to mark the keratin intermediate filaments and the cytoplasmic microtubules, respectively.

### 4.3. Fluid Flow Assay

Embryos were anesthetized using 0.01% benzocaine in 0.1× MMR and placed on wells carved on PDMS-covered slides. Fluorescent beads were placed in the well, and 15 s movies were recorded using Zeiss Axioimager (Carl Zeiss Microscopy GmbH, Jena, Germany). Movies were analyzed using IMARIS particle tracking analysis.

### 4.4. Immunostaining

Embryos were fixed at the appropriate developmental stage in MEMFA (10X: 1 M MOPS, 20 mM EGTA, 10 mM MgSO_4_, 38% formaldehyde) for 2 h at room temperature or overnight at 4 °C. Fixed embryos were permeabilized in PBT (0.5% Triton X-100, 1% DMSO in PBS) for two hours at room temperature, blocked for 30 min in 10% (*v*/*v*) donkey serum in PBT, and incubated with primary antibodies overnight at 4 °C. After several washes in PBT, embryos were stained with secondary antibodies (Alexa fluor conjugated, Invitrogen 1:250, phalloidin-647 plus, Invitrogen 1:1000) for 2 h at room temperature, washed several times in PBT, and fixed in MEMFA for 10 min. Primary antibodies: anti-acetylated tubulin (Santa Cruz, 611b1 clone, 1:1000) and anti-β catenin (#51067-2-AP, Proteintech 1:1000), anti-CEP164 (#22227-1-AP, Proteintech, 1:1000).

### 4.5. Image Analysis and Quantifications

Live imaging of tadpoles and imaging of fixed embryos was performed on Zeiss LSM700 or LSM900 with airyscan2 laser scanning confocal microscopes (Carl Zeiss Microscopy GmbH, Jena, Germany). For live imaging, embryos were placed on silicone grease wells on glass slides and anesthetized using 0.01% benzocaine in 0.1× MMR.

All quantifications were performed using ΙmageJ [48]. The orthogonal projections were generated using ΙmageJ, and final figures were prepared using Photoshop, version 26.2. Quantification of cell surface area was based on phalloidin staining of cortical actin.

Quantification of the DSP fluorescence intensity was performed using ImageJ version 1.54p. The same ROI was used across cells to quantify the mean fluorescence intensity in the cytosol, across the apical cell membrane, and at the rootlet. The mean fluorescence intensity in each cell was normalized against the cytosolic GFP levels to account for variations in expression levels across different cells.

### 4.6. Statistical Analysis

Significance was determined with a Student’s *t*-test (Figure 4B,D–F, Figure 5B, Figure 6F,G and Figure 8B,C), chi-square test (Figure 5D–F and Figure 6B), or one-way ANOVA (Figure 6D). All experiments were performed in duplicates, and the total number of cells and embryos quantified for each analysis is indicated in the figure legend. All bar graphs represent the mean, and error bars indicate the standard error of the mean, statistical analyses, ns: not significant, ** *p* < 0.01, and *** *p* < 0.001.

## Data Availability

Reagents used in this study are available on request from the corresponding authors.

## Supplementary Materials

The following supporting information can be downloaded at https://www.mdpi.com/article/10.3390/ijms26115381/s1.

## Abbreviations

The following abbreviations are used in this manuscript:

MCC	Multiciliated cell
PKP3	Plakophilin 3
DSP	Desmoplakin
CA	Ciliary adhesions
