# Supplementary material for: Plakophilin 3 Is Involved in Basal Body Docking in Multiciliated Cells
Source: Int J Mol Sci. 2025 Jun 4;26(11):5381. doi: 10.3390/ijms26115381 (PMC12155070; doi:10.3390/ijms26115381)
Supplement: Supplementary file 1 [file ijms-26-05381-s001.zip › ijms-3570563-supplementary.pdf]

Supplementary Figures

Figure S1. Localization of Xenopus PKP3-GFP in MCCs. MIP confocal image of a MCC that expresses PKP3-GFP and centrin-RFP. The graph shows the fluorescence intensity for centrin-RFP and PKP3-GFP along the white line in the zoomed in image.

Figure S2. Multiple and pairwise sequence alignment and of Xenopus and Human PKP3. Multiple sequence alignment was performed using Clustal Omega tool and default settings. Pairwise sequence alignment was performed using EMBOSS-needle and default settings. The following input sequences were used: H.s PKP3a- NP_009114.1, H.s PKP3b- NP_001289958.1, X.l PKP3L- NP_001086592.1, X.l PKP3S- NP_001084424.1. Description of the conservation score symbols: * (asterisk) shows conserved residues,: (colon) shows conservation between groups of strongly similar properties, . (period) shows conservation between groups of weakly similar properties,( ) shows a non conservative mutation, - shows gaps in sequence.

Figure S3: PKP3-GFP expression levels do not affect the localization of PKP3 near basal bodies. MIP confocal images of MCCs that express low (A) and high levels (B) of PKP3-GFP. The images were acquired under the same settings and differences in fluorescence intensity reflect differences in protein levels. The zoomed image in panel B shows a MCC that expresses low levels of PKP3-GFP. This cell is next to a MCC that expresses high levels of PKP3-GFP. In both cells PKP3-GFP localizes near basal bodies.

Figure S4. Sequence alignment of Xenopus and Human DSP. Sequence alignment was performed using the EMBOSS needle pairwise Sequence Alignment tool and default settings. The following input sequences were used: H.s NP_001008844.1 and X.l XP_018123268.1. Description of the conservation score symbols: * (asterisk) shows conserved residues,: (colon) shows conservation between groups of strongly similar properties, . (period) shows conservation between groups of weakly similar properties,( ) shows a non conservative mutation, - shows gaps in sequence.

Figure S5: The organization of the actin and keratin8 network in *Xenopus* MCCs. (A) Super-resolution confocal image of a MCC expressing mRuby-keratin8 and stained with phalloidin. The graphs show the fluorescence intensity for keratin8 and phalloidin along the red rectangle shown in the apical and subapical image of phalloidin. (B) Orthogonal projections of the same MCCs as shown in (A).

Figure S6: Downregulation of PKP3 does not affect the localization of clamp. Embryos were injected at the 4-cell stage with clamp-GFP and at the 16-cell stage with the PKP3 morpholino and centrin-RFP. Orthogonal projections of confocal images of control and morphant MCCs from the epidermis of a mosaic morphant embryo shows that clamp-GFP localizes near basal bodies in morphants and projects basally.

Figure S7: The background signal of CEP164 antibody staining. (A) MIP confocal image of the epidermis of a mosaic morphant embryo stained with anti-CEP164 (red) and phalloidin. (B) zoomed in image of the area in (A) marked by a white rectangle and stacks of side views showing the CEP164 signal and phalloidin in a non-MCC and a MCC. The outline of the MCC is marked by a white line.

Table S1: Putative PKP3 interactors with known functions in centriole and cilia biology

Movie S1. High-speed video of fluorescent beads as they move across the surface of a control embryos.

Movie S2. High-speed video of fluorescent beads as they move across the surface of a morphant embryos.

Figure S1

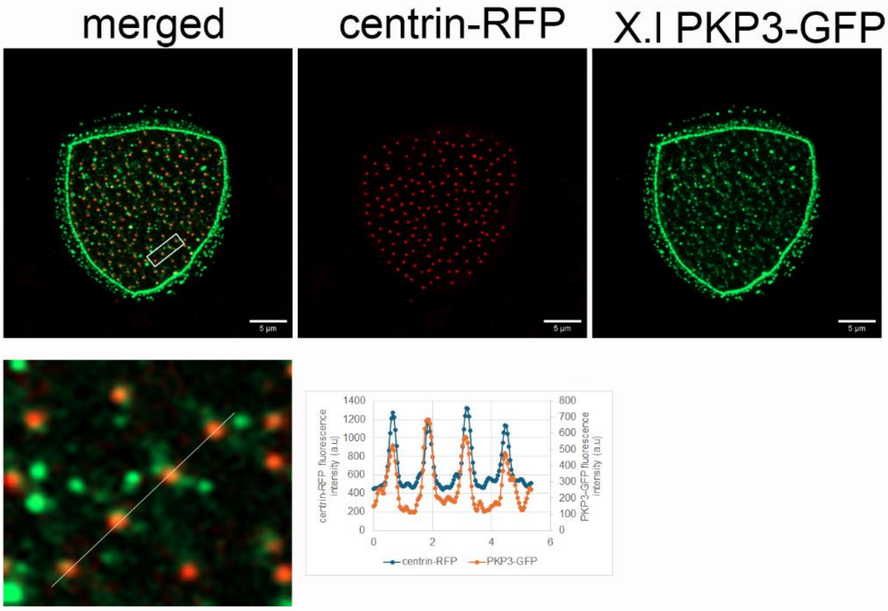
merged
centrin-RFP
X.l PKP3-GFP
5 µm
5 µm
5 µm
centrin-RFP fluorescence intensity (a.u)
PKP3-GFP fluorescence intensity (a.u)
centrin-RFP
PKP3-GFP

Figure S2

CLUSTAL O(1.2.4) multiple sequence alignment

```
H.s_PKP3a     --------------MQDGNFLLSALQPEAGVCSLALPSDLQLDRRGAEGPEAERLRAAR     45
H.s_PKP3b     MESWTPRPSAVASGMSWEAGGIRTTSRPEAGVCSLALPSDLQLDRRGAEGPEAERLRAAR
       60
X.l_PKP3L     --------------MQESHFLMSVLQPYTDISSLALPTDSQMDRRMREE--SVTLRNAR     43
X.l_PKP3S     --------------MHENHFLMSALQPHNDITSLALPTDSQMDRRMREE--SLALRNAR     43
                            :  : :. .*  . *****.* *.***  *   :  ** **

H.s_PKP3a     VQEQVRARLLQLGQQPRHNGAAE-PEPEAETARGTSRGQYHTLQAGFSSRSQGLSGDK--  102
H.s_PKP3b     VQEQVRARLLQLGQQPRHNGAAE-PEPEAETARGTSRGQYHTLQAGFSSRSQGLSGDK--  117
X.l_PKP3L     VQEQVRAKMTQKTPRGTGSNSSNNFSVSSYGDVFLPRTQYGSASASYSSRSQVLNSEPRM103
X.l_PKP3S     VQEQVRAKMMQKTQTTPRVSSNSNYSISSYGDTFMPRTQYGSASASYSSRSQVLSSEPKI  103
              *******:. *        .: . . .:      * ** . : .. .***** * .:.

H.s_PKP3a     ----TSGFRPIAKP--AYSPASWSSRSAVDLSCSRRLSSAHNGGSAFGAAGYGGAQPTPP  156
H.s_PKP3b     ----TSGFRPIAKP--AYSPASWSSRSAVDLSCSRRLSSAHNGGSAFGAAGYGGAQPTPP  171
X.l_PKP3L     SAVRTSGYSSV-----GGMRPGYSSRSAVDVGGNQRISVAS--------------QPRGY  144
X.l_PKP3S     STGRTSAYSVVGGGGGGGMRSGYSSRSAVDIGGTQRISVAS--------------QQRGY  149
                  **.: :     . ..:*******:.  .:*:.: *               *

H.s_PKP3a     MPTRPVSFHERGGVGSRADYDTLSLRSLRLGPGGLDDRYSLVS------EQLEPAATSTY  210
H.s_PKP3b     MPTRPVSFHERGGVGSRADYDTLSLRSLRLGPGGLDDRYSLVS------EQLEPAATSTY  225
X.l_PKP3L     TTSRPTSYHEKM-YPARGDYDSMSLRSLRIGDGEERYNQGSASQSSFYGRQMST---SSA  200
X.l_PKP3S     STSRPNSFHEKV-YPARRDYDTMSLRSLRIGDGEERYNQGPASQSGFYGRQVST---SSA  205
               .:** *:**:   .:* ***:.:******:* *     . . .*      .*:..   *:

H.s_PKP3a     RAFAYERQASSSSSRAGGLDWPEATEVSPSRTIRAPAVRTLQRFQSSHRSRGV-------  263
H.s_PKP3b     RAFAYERQASSSSSRAGGLDWPEATEVSPSRTIRAPAVRTLQRFQSSHRSRGV-------  278
X.l_PKP3L     GPMLQRSLSGNIGQDGGGSSWVERAEVAQTRTIRAPAMRTLQRFQTNNRNRVGSAPYGTI  260
X.l_PKP3S     GPVLQRSLSGNIGQNGGGTSWVERAEVAQTRTIRAPAMRTLQRFQSTNRARIGSAPYGTI  265
               .  .  :.:. .. . .** .* * .:**.:.:. .*******.*******. ..:.* *

H.s_PKP3a     --------------------------------GGAVPGAVLEPVARAPSVRSLSLSLAD290
H.s_PKP3b     --------------------------------GGAVPGAVLEPVARAPSVRSLSLSLAD305
X.l_PKP3L     NTGSQQQMQQMQQMQQMQQIQQMQQVQQSSGGIGSTYVTNMMEHNSRAPSVRSLAESGNQ
       320
X.l_PKP3S     NTGSQQQMQQMQQMQQ------MQQMQQSSSNMGSTYVTNMMGHNSRAPSVRSLAESGHQ
       319
                                          *.:   ::  :********. *  :

H.s_PKP3a     SGHLPDVHGFNSYGSH---RTLQRLSSGFDDIDLPSAVKYLMASDPNLQVLGAAYIQHKC  347
H.s_PKP3b     SGHLPDVHGFNSYGSH---RTLQRLSSGFDDIDLPSAVKYLMASDPNLQVLGAAYIQHKC  362
X.l_PKP3L     ---VQEIRGIDMFDGHKSLMSQHSFTSGFDDMDMPMAVKYLMASDANLQVLGAAYIQHRC  377
X.l_PKP3S     ---VQEIRGIDMFDGNKSLMSQHSFTSGFDDMDMPMAVKHLMASDPNLQVLGAAYIQHRC  376
                 : .:.*:.:.:..:.   : :. :.:*****.*.* ***.***** ************:*

H.s_PKP3a     YSDAAAKKQARSLQAVPRLVKLFNHANQEVQRHATGAMRNLIYDNADNKLALVEENGIFE  407
H.s_PKP3b     YSDAAAKKQARSLQAVPRLVKLFNHANQEVQRHATGAMRNLIYDNADNKLALVEENGIFE  422
X.l_PKP3L     YSDGEAKKQARSLQAIPKLVKLLNCDNQEVQRHATGAMRNLIYDNPENKMALVEENGIYE  437
X.l_PKP3S     YNDSEAKKQARSVQAIPKLVKLFNCDNQEVQRHATGAMRNLIYDNPENKMALVEENGIYE  436
              *.*. *******:**:*:****:* .*  ****************** .:**:********:*
```

```
H.s_PKP3a     LLRTLREQDDELRKNVTGILWNLSSSDHLKDRLARDTLEQLTDLVLSPLSGAGGPPLIQQ     467
H.s_PKP3b     LLRTLREQDDELRKNVTGILWNLSSSDHLKDRLARDTLEQLTDLVLSPLSGAGGPPLIQQ     482
X.l_PKP3L     LMTALEEPDDELRKNVTGILWNLSSSDNLKARLARDTLNQLTQRVLSPLSGTAGSAVIQQ     497
X.l_PKP3S     LLTALEEPDDELRKNVTGILWNLSSSDNLKARLARDTLNPLTQKVLSPLSGTAGSAVIQQ     496
              *. .* * ********************.** ********. **. *******. *  .***
               . . .                   .            .   .          ..   .

H.s_PKP3a     NASEAEIFYNATGFLRNLSSASQATRQKMRECHGLVDALVTSINHALDAGKCEDKSVENA     527
H.s_PKP3b     NASEAEIFYNATGFLRNLSSASQATRQKMRECHGLVDALVTSINHALDAGKCEDKSVENA     542
X.l_PKP3L     NVSEAEIFYNATGYLRNLSSASPETRQKMRECPGLLDSLVSYTNNALQSGKSEDKSVENV     557
X.l_PKP3S     NVSESEIFYNSTGFLRNLSSASPETRQRMRECPGLLDSLVSYISNALQSGKSEDKSVENA     556
              * **.*****.**.*********  ***.**** **.*.**.   .**..** *******
               .  .     .  .           .         . .  .  ..  ..  .      .

H.s_PKP3a     VCVLRNLSYRLYDEMPPSALQRLEGRGRRDLAGAPPGEVVGCFTPQSRRLRELP--LAAD     585
H.s_PKP3b     VCVLRNLSYRLYDEMPPSALQRLEGRGRRDLAGAPPGEVVGCFTPQSRRLRELP--LAAD     600
X.l_PKP3L     VCVLRNLSYRLYDEMPPSSLQRLEGGQRGR---TTAGDMVGCFTPQSRKLKEQQQQQGAD     614
X.l_PKP3S     VCVLRNLSYRLYDEMPPSSLQRLEGGQRGR---STVGDTVGCFTPQSRKLKEQQ-QQGAD     612
              ********************.******  *    .  *. *********.*.*     **
                                  .             .  .          . .     .

H.s_PKP3a     ALTFAEVSKDPKGLEWLWSPQIVGLYNRLLQRCELNRHTTEAAAGALQNITAGDRRWAGV     645
H.s_PKP3b     ALTFAEVSKDPKGLEWLWSPQIVGLYNRLLQRCELNRHTTEAAAGALQNITAGDRRWAGV     660
X.l_PKP3L     IATFTEISRDPKGMELLWHPQIVNLYNRLLQHCELNKYTTEAAAGALQNITAGDRRWASV     674
X.l_PKP3S     IATFTEISRDPKGMELLWHPQIVNLYNRLLQQCELNKYTTEAAAGALQNITAGDRRWASV     672
               **.*.*.****.* ** **** *******.****..********************** *
                 . . .    .        .        .    ..                     .

H.s_PKP3a     LSRLALEQERILNPLLDRVRTADHHQLRSLTGLIRNLSRNARNKDEMSTKVVSHLIEKLP     705
H.s_PKP3b     LSRLALEQERILNPLLDRVRTADHHQLRSLTGLIRNLSRNARNKDEMSTKVVSHLIEKLP     720
X.l_PKP3L     LSQVALDQERILNPVLDRLRTADHNQLRSLTGLIRNLSRHAKNKDEMSTKLVSHLLEKLP     734
X.l_PKP3S     LSQVALDQERILNPVLDRLRTADHNQLRSLTGLIRNLSRHAKNKDEMSTKLVSHLLEKLP     732
              **..**.*******.***.*****.***************.*.*********.****.****
                ..  .       .  .    .                .  .         .    .

H.s_PKP3a     GSVGEKSPPAEVLVNIIAVLNNLVVASPIAARDLLYFDGLRKLIFIKKKRDSPDSEKSSR     765
H.s_PKP3b     GSVGEKSPPAEVLVNIIAVLNNLVVASPIAARDLLYFDGLRKLIFIKKKRDSPDSEKSSR     780
X.l_PKP3L     ADSGEKCPPAEVLVNIIAILNNLTVAGPLAARDIAYFNGLSKLMYIKKMKDSPDSDKASR     794
X.l_PKP3S     ADSGEKSPPADVLVNIIAILNNLTTAGPLAARDIVYFNGLGKLMYIKKMKDSPDGDKAAR     792
                *** ***.********.****  * *.****. **.** **..*** .**** .*..*
              ..    .  .       .     .. . .    .   .    ..    .    .. ..

H.s_PKP3a     AASSLLANLWQYNKLHRDFRAKGYRKEDFLGP          797
H.s_PKP3b     AASSLLANLWQYNKLHRDFRAKGYRKEDFLGP          812
X.l_PKP3L     AACSLLTNMWQYSKLHREYKAKGYRKDDFLNP          826
X.l_PKP3S     AACSLLTNMWQYSKLHREYKAKGYRKDDFLSA          824
              ** ***.*.*** ****...******.***
                .    . .   .     ...       .    .
```

```
#
#
#  Percent Identity  Matrix - created by Clustal2.1
#
#

                          1       2       3       4
   1: H.s_PKP3a   100.00   98.49   62.50   61.61
   2: H.s_PKP3b    98.49  100.00   61.72   60.83
   3: X.l_PKP3L    62.50   61.72  100.00   89.62
   4: X.l_PKP3S    61.61   60.83   89.62  100.00
```

Pairwise sequence alignment using EMBOSS-needle

```
#=======================================
#
# Aligned_sequences: 2
# 1: PKP3L_Xenopus
# 2: PKP3a_HUMAN
# Matrix: EBLOSUM62
# Gap_penalty: 10.0
# Extend_penalty: 0.5
#
# Length: 870
# Identity:     497/870 (57.1%)
# Similarity:   611/870 (70.2%)
# Gaps:         123/870 (14.1%)
# Score: 2328.5
#
#
#=======================================

PKP3L_Xenopus      1 ------MRSAVIPTGQPQPYTDITSLALPTDSQMDRRMRE--ESVTLRNA
42
                           :.||:      ||...:.|||||:|.|:|||..|  |:..||.|
PKP3a_HUMAN        1 MQDGNFLLSAL------QPEAGVCSLALPSDLQLDRRGAEGPEAERLRAA
44

PKP3L_Xenopus     43 RVQEQVRAKMTQ--KTPRGTGSNSSNNFSVSSYGDVFLPRTQYGSASASY
90
                     ||||||||::.|  :.||..|:......:.::.|   ..|.||.:..|.:
PKP3a_HUMAN       45 RVQEQVRARLLQLGQQPRHNGAAEPEPEAETARG---TSRGQYHTLQAGF
91

PKP3L_Xenopus     91 SSRSQVLNSEPRMSAVRTSGYSSVGGMRPGY-----SSRSAVDVGGNQRI
135
                     |||||.|:.:      :|||:..:.  :|.|     |||||||:..::|:
PKP3a_HUMAN       92 SSRSQGLSGD------KTSGFRPIA--KPAYSPASWSSRSAVDLSCSRRL
133

PKP3L_Xenopus    136 SVA--------------SQPRGYTTSRPTSYHEK-MYPARGDYDSMSLRS
170
                     |.|              :||.....:||.|:||: ...:|.|||::||||
PKP3a_HUMAN      134 SSAHNGGSAFGAAGYGGAQPTPPMPTRPVSFHERGGVGSRADYDTLSLRS
183

PKP3L_Xenopus    171 LRIGDG--EERYNQGS-----ASQSSF----YGRQMSTSSAGPMLQRSLS
209
                     ||:|.|  ::||:..|     |:.|::    |.||.|:||:
PKP3a_HUMAN      184 LRLGPGGLDDRYSLVSEQLEPAATSTYRAFAYERQASSSSS---------
224

PKP3L_Xenopus    210 GNIGQDGGGSSWVERAEVAQTRTIRAPAMRTLQRFQTTNRNRVGSAPYGT
259
                          ..||..|.|..||:.:|||||||:|||||||:::|:|
PKP3a_HUMAN      225 -----RAGGLDWPEATEVSPSRTIRAPAVRTLQRFQSSHRSR--------
261

PKP3L_Xenopus    260 INTGSQQQMQQMQQIQQMQQLQQSSGGIGSTYVTNMMEHNSRAPSVR---
306
                                               |:|......::|..:||||||
```

```
PKP3a_HUMAN         262 --------------------------GVGGAVPGAVLEPVARAPSVRSLS
285

PKP3L_Xenopus       307 -SLAESGNQVQEIRGIDMFDGHKSLMSQHSFTSGFDDMDMPMAVKYLMAS
355
                         |||:||: :.::.|.:.:..|::|   ...:|||||:|:|.||||||||
PKP3a_HUMAN         286 LSLADSGH-LPDVHGFNSYGSHRTL---QRLSSGFDDIDLPSAVKYLMAS
331

PKP3L_Xenopus       356 DANLQVLGAAYIQHRCYSDGEAKKQARSLQAIPKLVKLLNCDNQEVQRHA
405
                        |.||||||||||||:||||..||||||||||:|:||||.|..||||||||
PKP3a_HUMAN         332 DPNLQVLGAAYIQHKCYSDAAAKKQARSLQAVPRLVKLFNHANQEVQRHA
381

PKP3L_Xenopus       406 TGAMRNLIYDNPENKMALVEENGIYELMTALEEPDDELRKNVTGILWNLS
455
                        |||||||||||.:||:||||||||:||:..|.|.||||||||||||||||
PKP3a_HUMAN         382 TGAMRNLIYDNADNKLALVEENGIFELLRTLREQDDELRKNVTGILWNLS
431

PKP3L_Xenopus       456 SSDNLKARLARDTLNQLTQRVLSPLSGTAGSAVIQQNVSEAEIFYNATGY
505
                        |||:||.|||||||.|||..|||||||..|..:||||.||||||||||||:
PKP3a_HUMAN         432 SSDHLKDRLARDTLEQLTDLVLSPLSGAGGPPLIQQNASEAEIFYNATGF
481

PKP3L_Xenopus       506 LRNLSSASPETRQKMRECPGLLDSLVSYTNNALQSGKSEDKSVENVVCVL
555
                        ||||||||..||||||||.||:|:||:..|:||.:||.|||||||.||||
PKP3a_HUMAN         482 LRNLSSASQATRQKMRECHGLVDALVTSINHALDAGKCEDKSVENAVCVL
531

PKP3L_Xenopus       556 RNLSYRLYDEMPPSSLQRLEGGQRGR-----TTAGDMVGCFTPQSRKLKE
600
                        ||||||||||||||:||||||  |||     ...|::||||||||||:|:|
PKP3a_HUMAN         532 RNLSYRLYDEMPPSALQRLEG--RGRRDLAGAPPGEVVGCFTPQSRRLRE
579

PKP3L_Xenopus       601 QQQQQGADIATFTEISRDPKGMELLWHPQIVNLYNRLLQHCELNKYTTEA
650
                          ....||..||.|:|:||||:|.||.||||.|||||||.||||::||||
PKP3a_HUMAN         580 --LPLAADALTFAEVSKDPKGLEWLWSPQIVGLYNRLLQRCELNRHTTEA
627

PKP3L_Xenopus       651 AAGALQNITAGDRRWASVLSQVALDQERILNPVLDRLRTADHNQLRSLTG
700
                        ||||||||||||||||.|||::||:|||||||:|||:|||||:|||||||
PKP3a_HUMAN         628 AAGALQNITAGDRRWAGVLSRLALEQERILNPLLDRVRTADHHQLRSLTG
677

PKP3L_Xenopus       701 LIRNLSRHAKNKDEMSTKLVSHLLEKLPADSGEKCPPAEVLVNIIAILNN
750
                        |||||||:|:||||||||:||||:||||...|||.|||||||||||:|||
PKP3a_HUMAN         678 LIRNLSRNARNKDEMSTKVVSHLIEKLPGSVGEKSPPAEVLVNIIAVLNN
727
```

```
PKP3L_Xenopus     751 LTVAGPLAARDIAYFNGLSKLMYIKKMKDSPDSDKASRAACSLLTNMWQY
800
                      |.||.|:||||:.||:||.||::|||.:|||||:|:||||.|||.|:|||
PKP3a_HUMAN       728 LVVASPIAARDLLYFDGLRKLIFIKKKRDSPDSEKSSRAASSLLANLWQY
777

PKP3L_Xenopus     801 SKLHREYKAKGYRKDDFLNP    820
                      :||||:::||||||:|||.|
PKP3a_HUMAN       778 NKLHRDFRAKGYRKEDFLGP    797


#---------------------------------------
#---------------------------------------
```

```
#=======================================
#
# Aligned_sequences: 2
# 1: PKP3S_Xenopus
# 2: PKP3a_HUMAN
# Matrix: EBLOSUM62
# Gap_penalty: 10.0
# Extend_penalty: 0.5
#
# Length: 865
# Identity:     499/865 (57.7%)
# Similarity:   615/865 (71.1%)
# Gaps:         109/865 (12.6%)
# Score: 2325.5
#
#
#=======================================

PKP3S_Xenopus      1 MHENHFLMSALQPHNDITSLALPTDSQMDRRMRE--ESLALRNARVQEQV
48
                     |.:.:||:|||||...:.|||||:|.|:|||..|  |:..||.|||||||
PKP3a_HUMAN        1 MQDGNFLLSALQPEAGVCSLALPSDLQLDRRGAEGPEAERLRAARVQEQV
50

PKP3S_Xenopus     49 RAKMMQKTQTTPRVSSNSNYSISSYGDTFMPRTQYGSASASYSSRSQVLS
98
                     ||:::|..| .||.:..:....:........|.||.:..|.:|||||.||
PKP3a_HUMAN       51 RARLLQLGQ-QPRHNGAAEPEPEAETARGTSRGQYHTLQAGFSSRSQGLS
99

PKP3S_Xenopus     99 SEPKISTGR---TSAYSVVGGGGGGGMRSGYSSRSAVDIGGTQRISVASQ
145
                     .: |.|..|   ..|||          .:.:|||||||:..::|:|.|..
PKP3a_HUMAN      100 GD-KTSGFRPIAKPAYS----------PASWSSRSAVDLSCSRRLSSAHN
138

PKP3S_Xenopus    146 ------QRGYS--------TSRPNSFHEK-VYPARRDYDTMSLRSLRIGD
180
                           ..||.        .:||.||||: ...:|.||||:||||||:|.
PKP3a_HUMAN      139 GGSAFGAAGYGGAQPTPPMPTRPVSFHERGGVGSRADYDTLSLRSLRLGP
188

PKP3S_Xenopus    181 G--EERYN-----QGPASQSGF----YGRQVSTSSAGPVLQRSLSGNIGQ
219
                     |  ::||:     ..||:.|.:    |.||.|:||:
PKP3a_HUMAN      189 GGLDDRYSLVSEQLEPAATSTYRAFAYERQASSSSS--------------
224

PKP3S_Xenopus    220 NGGGTSWVERAEVAQTRTIRAPAMRTLQRFQSTNRAR-IGSAPYGTINTG
268
                     ..||..|.|..||:.:|||||||:||||||||::|:| :|.|..|.:
PKP3a_HUMAN      225 RAGGLDWPEATEVSPSRTIRAPAVRTLQRFQSSHRSRGVGGAVPGAV---
271

PKP3S_Xenopus    269 SQQQMQQMQQMQQMQQMQQSSSNMGSTYVTNMMGHNSRAPSVR----SLA
314
                                                    :...:|||||||    |||
```

```
PKP3a_HUMAN      272 --------------------------------LEPVARAPSVRSLSLSLA
289

PKP3S_Xenopus    315 ESGHQVQEIRGIDMFDGNKSLMSQHSFTSGFDDMDMPMAVKHLMASDPNL
364
                     :||| :.::.|.:.:..:::|   ...:|||||:|:|.|||:||||||||
PKP3a_HUMAN      290 DSGH-LPDVHGFNSYGSHRTL---QRLSSGFDDIDLPSAVKYLMASDPNL
335

PKP3S_Xenopus    365 QVLGAAYIQHRCYNDSEAKKQARSVQAIPKLVKLFNCDNQEVQRHATGAM
414
                     ||||||||||:||:|:.|||||||:||:|:||||||..||||||||||||
PKP3a_HUMAN      336 QVLGAAYIQHKCYSDAAAKKQARSLQAVPRLVKLFNHANQEVQRHATGAM
385

PKP3S_Xenopus    415 RNLIYDNPENKMALVEENGIYELLTALEEPDDELRKNVTGILWNLSSSDN
464
                     |||||||.:||:||||||||:|||..|.|.|||||||||||||||||||:
PKP3a_HUMAN      386 RNLIYDNADNKLALVEENGIFELLRTLREQDDELRKNVTGILWNLSSSDH
435

PKP3S_Xenopus    465 LKARLARDTLNPLTQKVLSPLSGTAGSAVIQQNVSESEIFYNSTGFLRNL
514
                     ||.|||||||..||..|||||||..|..:||||.||:|||||:|||||||
PKP3a_HUMAN      436 LKDRLARDTLEQLTDLVLSPLSGAGGPPLIQQNASEAEIFYNATGFLRNL
485

PKP3S_Xenopus    515 SSASPETRQRMRECPGLLDSLVSYISNALQSGKSEDKSVENAVCVLRNLS
564
                     ||||..|||:||||.||:|:||:.|::||.:||.||||||||||||||||
PKP3a_HUMAN      486 SSASQATRQKMRECHGLVDALVTSINHALDAGKCEDKSVENAVCVLRNLS
535

PKP3S_Xenopus    565 YRLYDEMPPSSLQRLEGGQRGRSTV-----GDTVGCFTPQSRKLKEQQQQ
609
                     ||||||||||:||||||  |||..:     |:.|||||||||:|:| ...
PKP3a_HUMAN      536 YRLYDEMPPSALQRLEG--RGRRDLAGAPPGEVVGCFTPQSRRLRE-LPL
582

PKP3S_Xenopus    610 GADIATFTEISRDPKGMELLWHPQIVNLYNRLLQQCELNKYTTEAAAGAL
659
                     .||..||.|:|:||||:|.||.||||.|||||||:||||::|||||||||
PKP3a_HUMAN      583 AADALTFAEVSKDPKGLEWLWSPQIVGLYNRLLQRCELNRHTTEAAAGAL
632

PKP3S_Xenopus    660 QNITAGDRRWASVLSQVALDQERILNPVLDRLRTADHNQLRSLTGLIRNL
709
                     |||||||||||.|||::||:|||||||:|||:|||||:||||||||||||
PKP3a_HUMAN      633 QNITAGDRRWAGVLSRLALEQERILNPLLDRVRTADHHQLRSLTGLIRNL
682

PKP3S_Xenopus    710 SRHAKNKDEMSTKLVSHLLEKLPADSGEKSPPADVLVNIIAILNNLTTAG
759
                     ||:|:||||||||:||||:||||...|||||||:|||||||:||||..|.
PKP3a_HUMAN      683 SRNARNKDEMSTKVVSHLIEKLPGSVGEKSPPAEVLVNIIAVLNNLVVAS
732
```

```
PKP3S_Xenopus    760 PLAARDIVYFNGLGKLMYIKKMKDSPDGDKAARAACSLLTNMWQYSKLHR
809
                     |:||||::||:||.||::|||.:||||.:|::|||.|||.|:|||:||||
PKP3a_HUMAN      733 PIAARDLLYFDGLRKLIFIKKKRDSPDSEKSSRAASSLLANLWQYNKLHR
782

PKP3S_Xenopus    810 EYKAKGYRKDDFLSA    824
                     :::||||||:|||..
PKP3a_HUMAN      783 DFRAKGYRKEDFLGP    797


#---------------------------------------
#---------------------------------------
```

Figure S3

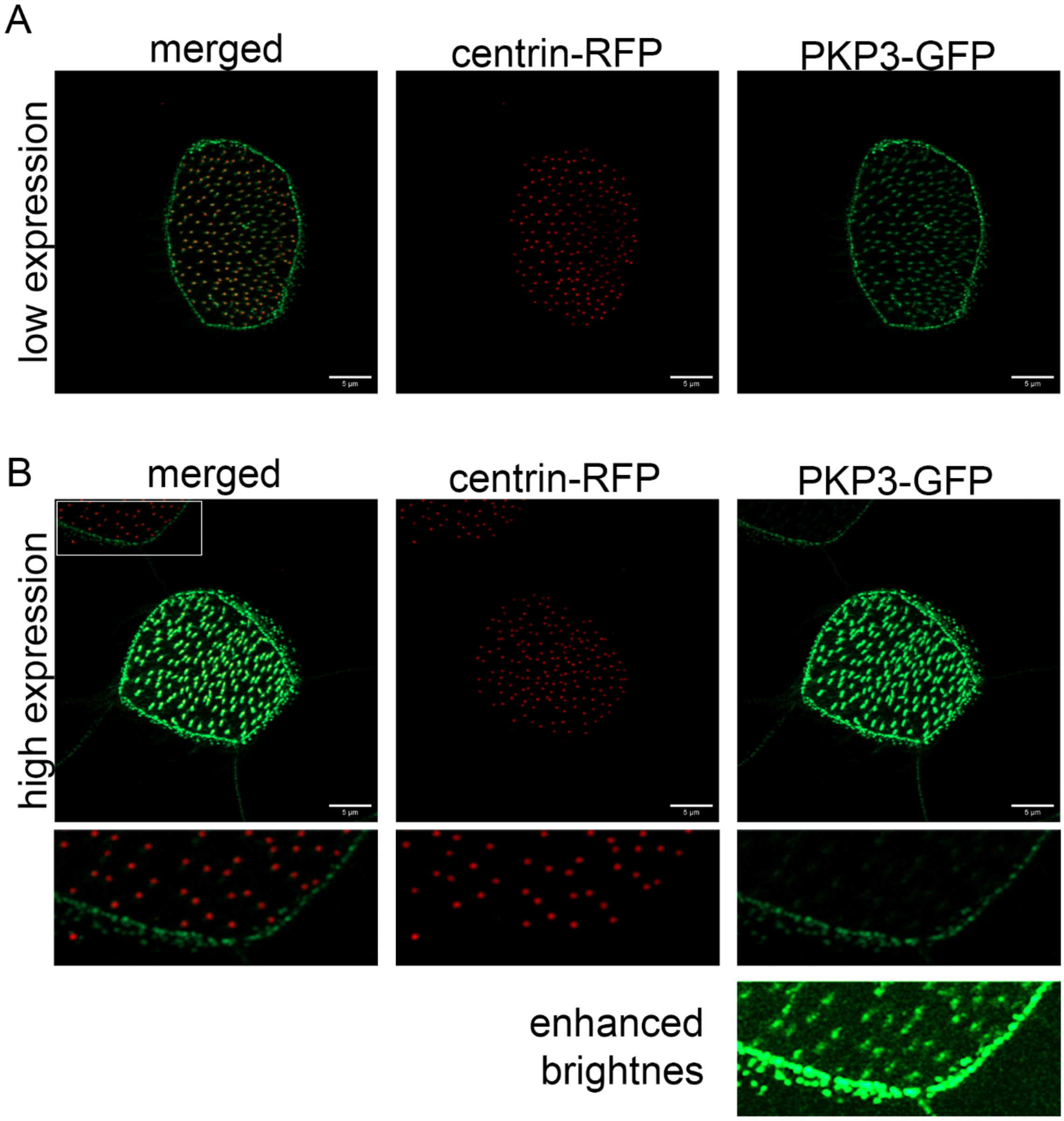
A
merged
centrin-RFP
PKP3-GFP
low expression
B
merged
centrin-RFP
PKP3-GFP
high expression
enhanced
brightnes

Figure S4

```
#=======================================
#
# Aligned_sequences: 2
# 1: H.s
# 2: Xl
# Matrix: EBLOSUM62
# Gap_penalty: 10.0
# Extend_penalty: 0.5
#
# Length: 2303
# Identity:    1546/2303 (67.1%)
# Similarity:  1899/2303 (82.5%)
# Gaps:          67/2303 ( 2.9%)
# Score: 7945.0
#
#
#=======================================

H.s                1 MSCNGGSHPRINTLGRMIRAESGPDL--RYEVTS------GGGGTSRMY-
41
                     ||.|||||.|||||||:.|:|||.|:  ||::.:      ||.|...|| 
Xl                 1 MSINGGSHTRINTLGRLTRSESGQDISGRYDMNNYHKMVGGGPGIQPMYT
50

H.s               42 ---YSRRGVITDQNSDGYCQTGTMSRHQNQNTIQELLQNCSDCLMRAELI
88
                        ||     ::...|.|.|.|||:|  ..||:.:|:||.|..|.:|||:
Xl                51 TTVYS-----SETTGDSYGQGGTMAR--RPNTVNDLIQNISANLGQAELV
93

H.s               89 VQPELKYGDGIQLTRSRELDECFAQANDQMEILDSLIREMRQMGQPCDAY
138
                     :|.||||||..|..|:|:|||....||:|:||:|.||||||.|||||:.|
Xl                94 IQHELKYGDASQPVRNRDLDEYLGVANEQIEIVDGLIREMRAMGQPCEQY
143

H.s              139 QKRLLQLQEQMRALYKAISVPRVRRASSKGGGGYTCQSGSGWDEFTKHVT
188
                     .::|.|||:|||||||||:.||:|:.|.  .||::.||||||||.||.:|
Xl               144 SRKLAQLQDQMRALYKAINGPRMRKGSK--SGGFSSQSGSGWDEHTKRIT
191

H.s              189 SECLGWMRQQRAEMDMVAWGVDLASVEQHINSHRGIHNSIGDYRWQLDKI
238
                     ||.||.:||.|.:::||.||.|.:||||.:.:||..||:|.||||.||||
Xl               192 SETLGQIRQHRRQLEMVDWGFDASSVEQQLANHRKFHNAIADYRWDLDKI
241

H.s              239 KADLREKSAIYQLEEEYENLLKASFERMDHLRQLQNIIQATSREIMWIND
288
                     |||:|||.|||||||||:.||||||||||.|||||.:|||||:|||||||
Xl               242 KADVREKGAIYQLEEEYDCLLKASFERMDQLRQLQAVIQATSKEIMWIND
291
```

```
H.s              289 CEEEELLYDWSDKNTNIAQKQEAFSIRMSQLEVKEKELNKLKQESDQLVL
338
                     .|||||::||||:||:|.:|||:||..||:||:|||||||||||||||::
Xl               292 HEEEELVFDWSDRNTDIPKKQESFSKLMSELEIKEKELNKLKQESDQLIM
341

H.s              339 NQHPASDKIEAYMDTLQTQWSWILQITKCIDVHLKENAAYFQFFEEAQST
388
                     |||||:|||||||||||||||||||||||||||||||||||||||:|||||
Xl               342 NQHPAADKIEAYMDTLQTQWSWILQITKCIDVHLKENAAYFQFFDEAQST
391

H.s              389 EAYLKGLQDSIRKKYPCDKNMPLQHLLEQIKELEKEREKILEYKRQVQNL
438
                     |.||..||::|||||..||:|.||.:|:..|:||||:|||.|||||||:|
Xl               392 ENYLVNLQETIRKKYISDKSMSLQRVLDMTKDLEKEKEKINEYKRQVQSL
441

H.s              439 VNKSKKIVQLKPRNPDYRSNKPIILRALCDYKQDQKIVHKGDECILKDNN
488
                     ||||||||||||||||:..|.::|:||||||||||::|||||||||:|.
Xl               442 VNKSKKIVQLKPRNPDYKREKTVVLKALCDYKQDQKVIHKGDECILKNNT
491

H.s              489 ERSKWYVTGPGGVDMLVPSVGLIIPPPNPLAVDLSCKIEQYYEAILALWN
538
                     :||||.||||||:||:||||.|||||||||||||:.||||:|||||.|||
Xl               492 QRSKWNVTGPGGLDMVVPSVSLIIPPPNPLAVDLATKIEQFYEAILVLWN
541

H.s              539 QLYINMKSLVSWHYCMIDIEKIRAMTIAKLKTMRQEDYMKTIADLELHYQ
588
                     |||||||||:|||||||||||||||.|||||||||||.|.:.|||:|||
Xl               542 QLYINMKSLISWHYCMIDIEKIRAMTFAKLKTMRQEDYQKVVTDLEIHYQ
591

H.s              589 EFIRNSQGSEMFGDDDKRKIQSQFTDAQKHYQTLVIQLPGYPQHQTVT--
636
                     ||:||||||.||||:||||:||||::||||||||||:|||.  |..|||
Xl               592 EFLRNSQGSNMFGDEDKRKMQSQFSEAQKHYQTLVVQLPS--QSTTVTER
639

H.s              637 ------TTEITHHGTCQDVNHNKV----IETNRENDKQETWMLMELQKIR
676
                           .|.||.. ..::|:.:.|    .:.:|:.:|||.|:|:|||:||
Xl               640 KMVQQNATPITKE-VVKNVSSSMVEVDSKQIDRDREKQENWLLLELQRIR
688

H.s              677 RQIEHCEGRMTLKNLPLADQGSSHHITVKINELKSVQNDSQAIAEVLNQL
726
                     :|:|..|.|:...|:...||.::.....:|:|:::::.:.|.||..::::
Xl               689 KQLEGYEIRLVRNNIYNVDQETAKDFPRRISEIETLKGEGQGIATQISKI
738

H.s              727 KDMLANFRGSEKYCYLQNEVFGLFQKLENINGVTDGYLNSLCTVRALLQA
776
                     |:|:.:.:.|:|..|||:|:..|.:|:|||:||.:..:|..|.::|.|||:
```

```
Xl                739 KEMILSLKESDKSSYLQSELSFLLKKMENVNGFSADHLERLQSLRLLLQS
788

H.s               777 ILQTEDMLKVYEARLTEEETVCLDLDKVEAYRCGLKKIKNDLNLKKSLLA
826
                      |||.||::||||.|||||||:.||..||||||..|||:|.:|..||.:|.
Xl                789 ILQIEDLIKVYEVRLTEEETISLDPAKVEAYRGTLKKMKMELEQKKGMLK
838

H.s               827 TMKTELQKAQQIHSQTSQQYPLYDLDLGKFGEKVTQLTDRWQRIDKQIDF
876
                      |:.:||:...||:.:.:|.||.|.||:.||..|..||.:|.|||:|:||.
Xl                839 TLDSELKSTLQINDRVNQAYPFYGLDMSKFWNKGNQLIERSQRIEKEIDD
888

H.s               877 RLWDLEKQIKQLRNYRDNYQAFCKWLYDAKRRQDSLESMKFGDSNTVMRF
926
                      |||:||||.||::||:|..|...||:.|.|.:||||||:|..|:.||.|.
Xl                889 RLWELEKQSKQIKNYKDVSQVLSKWISDTKHKQDSLESIKLSDATTVSRC
938

H.s               927 LNEQKNLHSEISGKRDKSEEVQKIAELCANSIKDYELQLASYTSGLETLL
976
                      :::||.|::||.|||||.|:|.|.|::||:.|||||||||||:|||||||
Xl                939 ISDQKALNAEIQGKRDKVEDVVKTADICASGIKDYELQLASYSSGLETLL
988

H.s               977 NIPIKRTMIQSPSGVILQEAADVHARYIELLTRSGDYYRFLSEMLKSLED
1026
                      ||||||||:|||||||.||:|::.|||||||||..||||.|.|..|||||
Xl                989 NIPIKRTMVQSPSGVITQESAEIQARYIELLTRGNDYYRLLHETSKSLED
1038

H.s              1027 LKLKNTKIEVLEEELRLARDANSENCNKNKFLDQNLQKYQAECSQFKAKL
1076
                      ||:|||:||:|||||||||||||:|..|||||||||||||.||::||:::
Xl               1039 LKMKNTRIELLEEELRLARDANSDNSQKNKFLDQNLQKYQIECNEFKSRI
1088

H.s              1077 ASLEELKRQAELDGKSAKQNLDKCYGQIKELNEKITRLTYEIEDEKRRRK
1126
                      ..||::||::|:||.||||||:|.|.||.|||:|||||||||:|||||||
Xl               1089 LLLEDMKRKSEMDGNSAKQNLEKYYSQITELNDKITRLTYEIDDEKRRRK
1138

H.s              1127 SVEDRFDQQKNDYDQLQKARQCEKENLGWQKLESEKAIKEKEYEIERLRV
1176
                      ::|||.|||||::||:||.:|.:.::|..||||:||..||||||||||::
Xl               1139 ALEDRCDQQKNEFDQMQKKKQNDLDSLNRQKLETEKIFKEKEYEIERLKI
1188

H.s              1177 LLQEEGTRKREYENELAKASNRIQESKNQCTQVVQERESLLVKIKVLEQD
1226
                      |||:||.|||||||||:|||::|.||.::.:::|||:||||.|||:||||
Xl               1189 LLQDEGQRKREYENELSKASSKIHESSSKWSEIVQEKESLLTKIKLLEQD
1238
```

```
H.s              1227 KARLQRLEDELNRAKSTLEAETRVKQRLECEKQQIQNDLNQWKTQYSRKE 1276
                      |.||||||:||||||:.|::|.|.|||||.||.||:||.||||:|::|||
Xl               1239 KIRLQRLEEELNRAKAALDSEIRNKQRLEEEKNQIRNDFNQWKSQFTRKE 1288

H.s              1277 EAIRKIESEREKSEREKNSLRSEIERLQAEIKRIEERCRRKLEDSTRETQ 1326
                      :.:::||.:.|:..|:|..|:||||||||||:.||||.||||:|:.|..|
Xl               1289 DDVKRIEIDLERCGRDKLGLKSEIERLQAEIRSIEERYRRKLDDANRTCQ 1338

H.s              1327 SQLETERSRYQREIDKLRQRPYGSHRETQTECEWTVDTSKLVFDGLRKKV 1376
                      |:::::|...:.|::.|::||.||.::|||:.:.::|.||.:||||||||
Xl               1339 SEMKSQRLALESELENLKRRPLGSVKQTQTDEDMSIDPSKFLFDGLRKKV 1388

H.s              1377 TAMQLYECQLIDKTTLDKLLKGKKSVEEVASEIQPFLRGAGSIAGASASP 1426
                      ||.||.|||:|.|.|.:||:||:||:||||:|::|:|:|||:|||.|.:|
Xl               1389 TAHQLLECQIITKATFEKLIKGQKSLEEVAAEVEPYLKGAGAIAGVSVTP 1438

H.s              1427 KEKYSLVEAKRKKLISPESTVMLLEAQAATGGIIDPHRNEKLTVDSAIAR 1476
                      ||||||||||||||||||||||||:||||||||||..:|||||.|.|:||
Xl               1439 KEKYSLVEAKRKKLISPESTVMLLQAQAATGGIIDIGKNEKLTADHALAR 1488

H.s              1477 DLIDFDDRQQIYAAEKAITGFDDPFSGKTVSVSEAIKKNLIDRETGMRLL 1526
                      |||::.|::.||.||||||||.||||||.|.|.||||||||.:..|:|||
Xl               1489 DLIEYSDKEAIYTAEKAITGFKDPFSGKIVGVGEAIKKNLISQSDGIRLL 1538

H.s              1527 EAQIASGGVVDPVNSVFLPKDVALARGLIDRDLYRSLNDPRDSQKNFVDP 1576
                      |||:|:||:|||:.||::|.||||:|||||::||:.|||..|..|||:||
Xl               1539 EAQVAAGGIVDPLISVYIPVDVALSRGLIDQNLYKILNDLSDDVKNFIDP 1588

H.s              1577 VTKKKVSYVQLKERCRIEPHTGLLLLSVQKRSMSFQGIRQPVTVTELVDS 1626
                      ||||.:|||||:::||:|||||.|||.|.|:|:||||||..|.|.|||.|
Xl               1589 VTKKNISYVQLRKKCRVEPHTGFLLLPVMKKSLSFQGIRHQVPVDELVQS 1638

H.s              1627 GILRPSTVNELESGQISYDEVGERIKDFLQGSSCIAGIYNETTKQKLGIY 1676
                      ||:|..||::||||||:.:||.|||||||||||||||||||.||:|||:.
Xl               1639 GIIRADTVDQLESGQITMEEVSERIKDFLQGSSCIAGIYNEATKEKLGLC 1688

H.s              1677 EAMKIGLVRPGTALELLEAQAATGFIVDPVSNLRLPVEEAYKRGLVGIEF 1726
                      :||..||:|.||.|||||||||||||||||:|||||||||::||||..||
```

```
Xl              1689 DAMNKGLLRRGTTLELLEAQAATGFIVDPVNNLRLPVEEAHRRGLVAHEF
1738

H.s             1727 KEKLLSAERAVTGYNDPETGNIISLFQAMNKELIEKGHGIRLLEAQIATG
1776
                     |:||||||||||||.|||||||||||||||.|||||||||||||||||||
Xl              1739 KDKLLSAERAVTGYKDPETGNIISLFQAMKKELIEKGHGIRLLEAQIATG
1788

H.s             1777 GIIDPKESHRLPVDIAYKRGYFNEELSEILSDPSDDTKGFFDPNTEENLT
1826
                     ||||||||||||||.|||||:||:|::||||:|.||||||||||||||||
Xl              1789 GIIDPKESHRLPVDAAYKRGFFNQEMNEILSNPDDDTKGFFDPNTEENLT
1838

H.s             1827 YLQLKERCIKDEETGLCLLPLKEKKKQVQTSQKNTLRKRRVVIVDPETNK
1876
                     |||||:|||.||:|.|||||||||||.|||||||||||||||||||:||:
Xl              1839 YLQLKDRCIVDEKTNLCLLPLKEKKKAVQTSQKNTLRKRRVVIVDPDTNR
1888

H.s             1877 EMSVQEAYKKGLIDYETFKELCEQECEWEEITITGSDGSTRVVLVDRKTG
1926
                     ||:||||||||||||.|:.||..||||||||||||||||:|:||||||:|
Xl              1889 EMTVQEAYKKGLIDYNTYIELSGQECEWEEITITGSDGSSRIVLVDRKSG
1938

H.s             1927 SQYDIQDAIDKGLVDRKFFDQYRSGSLSLTQFADMISLKNGVGTSSSMGS
1976
                     :|||:|:||:|||::|...||||:|||||||||||||..|
Xl              1939 NQYDVQEAIEKGLINRSQLDQYRAGSLSLTQFADMISNIN----------
1978

H.s             1977 GVSDDVFSSSRHESVS--KISTISSVRNLTIRSSSFSDTLEESSPIAAIF
2024
                      :||||..||||:|:|  ::.|.|     ..:|.|:||||||:|||||||
Xl              1979 -LSDDVLVSSRHDSLSSPRLRTSS-----WSKSGSYSDTLEETSPIAAIF
2022

H.s             2025 DTENLEKISITEGIERGIVDSITGQRLLEAQACTGGIIHPTTGQKLSLQD
2074
                     ||||:|||||:|||.|||||:|||||||||||||||||:|.|||:|||||
Xl              2023 DTENIEKISISEGINRGIVDTITGQRLLEAQACTGGIINPATGQRLSLQD
2072

H.s             2075 AVSQGVIDQDMATRLKPAQKAFIGFEGVKGKKKMSAAEAVKEKWLPYEAG
2124
                     ||:||:||.||::|||.||||:.||:|::|:.|:|||||:|..|||||||
Xl              2073 AVTQGIIDHDMSSRLKQAQKAYYGFDGIRGQTKLSAAEAMKVNWLPYEAG
2122

H.s             2125 QRFLEFQYLTGGLVDPEVHGRISTEEAIRKGFIDGRAAQRLQDTSSYAKI
2174
                     ||||||||:|||||||:..||:|||||||||.|||||||:|:|.:||.||
Xl              2123 QRFLEFQYITGGLVDPDTQGRLSTEEAIRKGMIDGRAAQKLRDVNSYPKI
2172
```

```
H.s            2175 LTCPKTKLKISYKDAINRSMVEDITGLRLLEAASVSSKGLPSPYNMSSAP
2224
                    |||||||||||||:|:::|||||.|.||:||||||||||:.|||||||||
Xl             2173 LTCPKTKLKISYKEAVDKSMVEDKTSLRMLEAASVSSKGISSPYNMSSAP
2222

H.s            2225 GSRSGSRSGSRSGSRSGSRSGSRRGSFDA-TGNSSYSYSYS---FSSSSI
2270
                             ||||||||||||||||||||| :|:||.|||:|   |||||.
Xl             2223 --------GSRSGSRSGSRSGSRRGSFDASSGSSSVSYSFSQNIFSSSSS
2264

H.s            2271 GH-    2272
                    |.
Xl             2265 GGI    2267


#---------------------------------------
#---------------------------------------
```

Figure S5

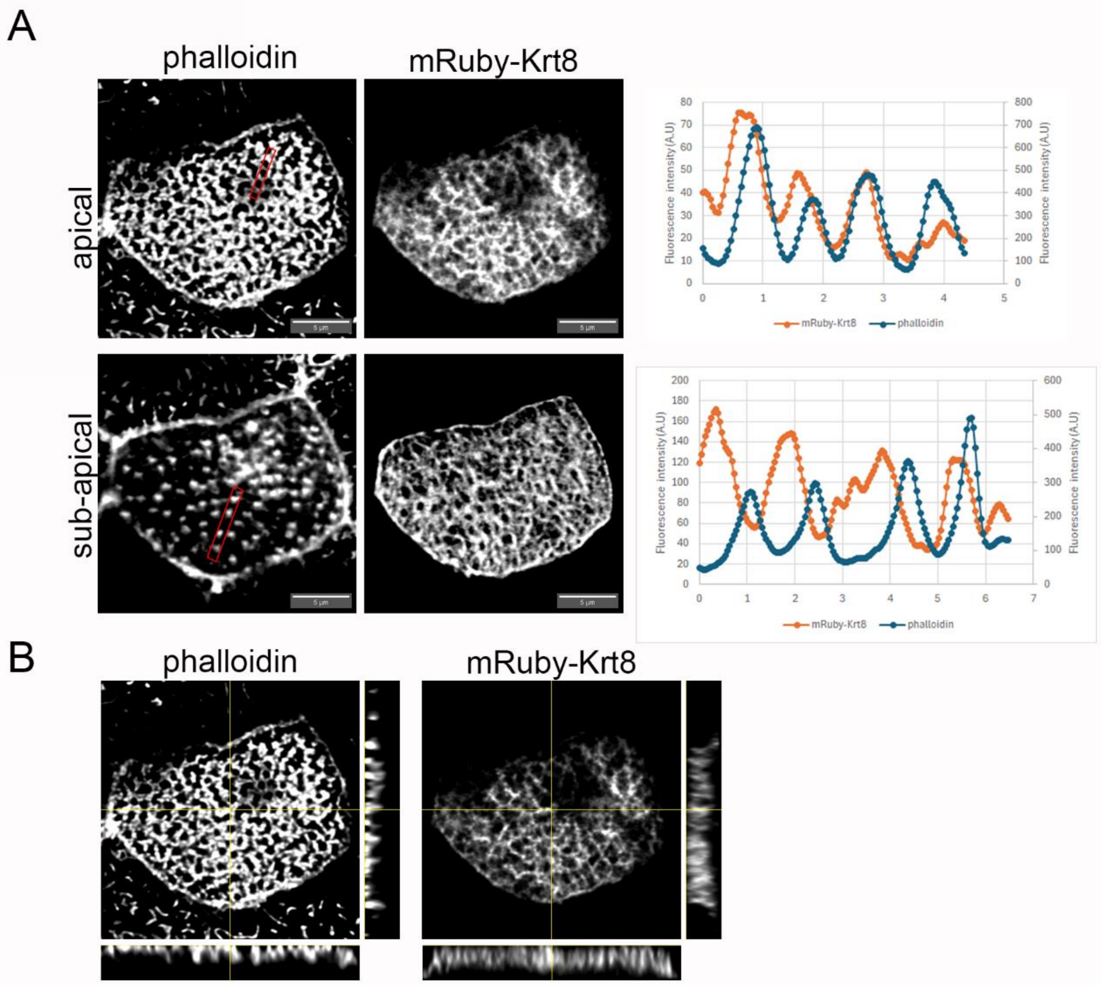
A
phalloidin
mRuby-Krt8
apical
sub-apical
Fluorescence intensity(A.U)
mRuby-Krt8
phalloidin
B
phalloidin
mRuby-Krt8

Figure S6

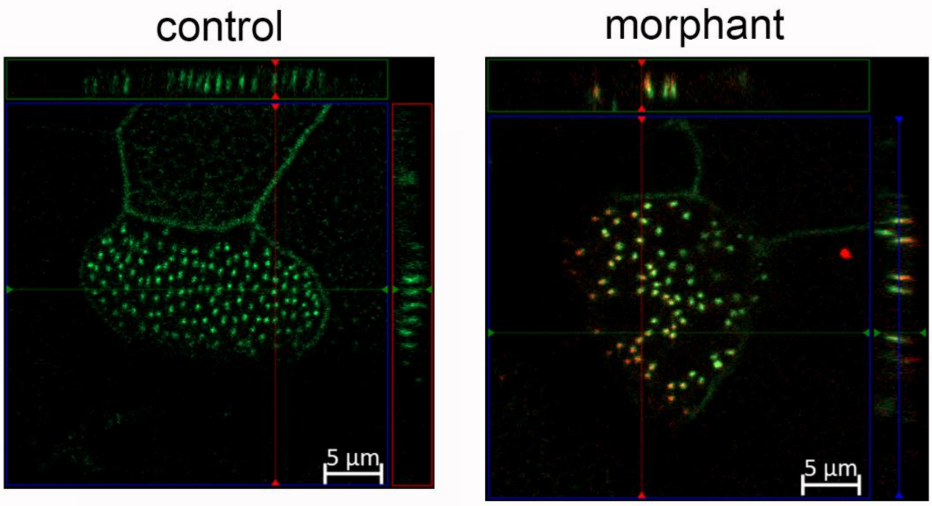

Figure S7

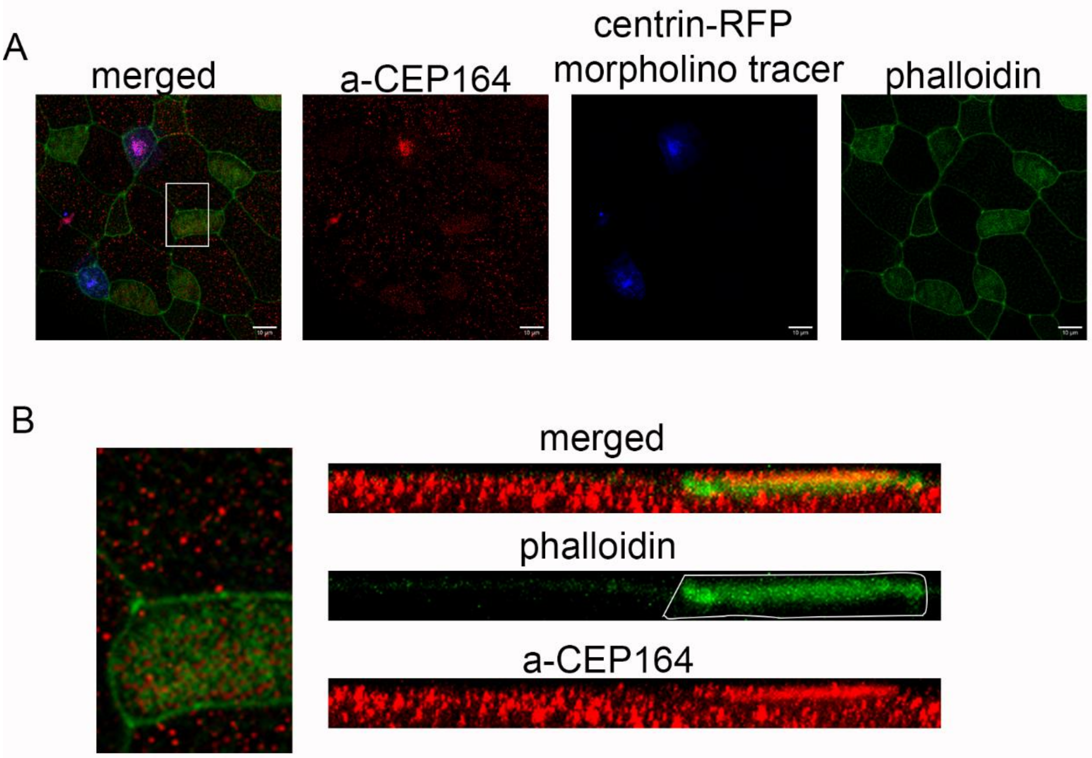
A
merged
a-CEP164
centrin-RFP
morpholino tracer
phalloidin
B
merged
phalloidin
a-CEP164

Table S1

**Table S1. Putative PKP3 interactors with known functions in centriole and cilia biology**					
#BioGRID Interaction ID	Entrez Gene Interactor A	Official Symbol Interactor A	Experimental System	Publication Source	Throughput
2245620	129881	CCDC173	Affinity Capture-MS	PUBMED:28514442	High Throughput
3331094	998	CDC42	Affinity Capture-MS	PUBMED:31871319	High Throughput
2561391	55755	CDK5RAP2	Affinity Capture-MS	PUBMED:29162697	High Throughput
2825901	128866	CHMP4B	Affinity Capture-MS	PUBMED:31586073	High Throughput
2472424	1499	CTNNB1	Affinity Capture-MS	PUBMED:27684187	High Throughput
3559860	1856	DVL2	Affinity Capture-MS	PUBMED:36398662	Low Throughput
2265919	55531	ELMOD1	Affinity Capture-MS	PUBMED:28514442	High Throughput
3202116	55294	FBXW7	Affinity Capture-MS	PUBMED:33658012	High Throughput
3069519	2805	GOT1	Affinity Capture-MS	PUBMED:33961781	High Throughput
2398107	3191	HNRNPL	Affinity Capture-RNA	PUBMED:28611215	High Throughput
2830238	9928	KIF14	Affinity Capture-MS	PUBMED:31586073	High Throughput
2229486	11127	KIF3A	Affinity Capture-MS	PUBMED:28514442	High Throughput
3389442	4000	LMNA	Proximity Label-MS	PUBMED:32344865	High Throughput
3054484	5601	MAPK9	Affinity Capture-MS	PUBMED:33961781	High Throughput

1105732	23031	MAST3	Affinity Capture-MS	PUBMED:25852190	High Throughput
2473103	9968	MED12	Affinity Capture-MS	PUBMED:27684187	High Throughput
2241155	9659	PDE4DIP	Affinity Capture-MS	PUBMED:28514442	High Throughput
3021750	11091	WDR5	Affinity Capture-MS	PUBMED:33022573	High Throughput
proteins with known function in MCCs					
